# Seasonal prevalence of extended-spectrum β-lactamase–producing bacteria in food-chain animals, humans, and the surrounding environment in Fayoum governorate: a one health approach

**DOI:** 10.3389/fmicb.2026.1726798

**Published:** 2026-02-04

**Authors:** Ayatollah S. El-Zayat, Shrouk E. Khalil, Marwa N. Ahmed, Dina El-Sayed, Neveen Rabie, Enas A. H. Farag, Hanan A. Goda, Ahmad F. Al-Shahaby, Hala R. Ali

**Affiliations:** 1 Department of Microbiology, Faculty of Agriculture, Cairo University, Giza, Egypt; 2 School of Biotechnology, Nile University, Giza, Egypt; 3 Reference Laboratory for Veterinary Quality Control on Poultry Production, Animal Health Research Institute, Agriculture Research Centre (ARC), Giza, Egypt; 4 Department of Pharmacology, Animal Health Research Institute, Agriculture Research Centre (ARC), Giza, Egypt; 5 Department of Bacteriology, Animal Health Research Institute (AHRI), Agriculture Research Centre (ARC), Giza, Egypt

**Keywords:** *bla* genes, *Escherichia coli*, extended-spectrum β-lactamase, *Klebsiella pneumoniae*, seasonal variation, β-lactam antibiotics

## Abstract

Extended-spectrum β-lactamase (ESBL) producers, particularly *Escherichia coli* and *Klebsiella pneumoniae*, pose a growing One Health challenge influenced by seasonal variation. This study assessed seasonal impacts on ESBL prevalence among humans, animals, and farm environments. A total of 2,890 poultry samples, 864 samples from dairy cows (including 88 milk samples and 776 rectal swabs), 248 human fecal samples (118 farm workers and 130 hospitalized patients) and 583 environmental samples were collected from Fayoum governorate, across three seasons. The isolation data revealed significant seasonal impacts, particularly in dairy cows and environmental samples, with source-related differences evident within the same season. The phenotypic and genotypic ESBL- analysis of all isolates from different sources across seasons showed that ESBL-producing *E. coli* demonstrated comparable prevalence in summer (14.68%) and fall (15%) before declining in winter (7.5%), while *K. pneumoniae* showed the highest prevalence in winter (29.4%), with lower detection in summer (11.9%) and absence in fall. Significant seasonal differences were detected, with ESBL*-*producing *E. coli* prevalence varying across sources in the fall (*p* = 0.039) and ESBL-producing *K. pneumoniae* showing variation in poultry across seasons (*p* = 0.042). Environmental isolates exhibited fluctuating trends, highlighting the role of farm environments in ESBL persistence and dissemination. At the genetic level, *blaSHV* and *blaCTX-M1* demonstrated seasonal variation, whereas *blaTEM* showed no variation. Heat-map and hierarchical clustering showed significant correlation among harboring ESBL-genes, particularly *blaSHV* and *blaCTX-M1*, and resistance profiles to β-lactams antibiotics, as well as to non-beta-lactam antibiotics. Additionally, source- and species-based seasonal effects were observed in the prevalence of *E. coli*, *K. pneumoniae*, and their associated ESBL traits. The results further demonstrated that genotypic resistance determinants (*bla* genes) are significantly linked to phenotypic resistance, especially to β-lactams, and also reflected multi-drug resistance patterns that indicate co-selection across unrelated antibiotic classes. These findings highlight the public health significance of ESBL-producing *E. coli* and *K. pneumoniae*, both as pathogens and as disseminators of multidrug resistance determinants, emphasizing the need for One Health surveillance. To the best of our knowledge, this is the first systematic and comprehensive investigation of ESBL prevalence across animal, human and environmental, over three distinct seasons.

## 1 Introduction

The rapid emergence and global spread of antimicrobial resistance in animals represent a critical challenge that pose a threat to the public health, livestock production, and environmental safety (Hamed et al., 2025; Fathy et al., 2025; Peirano and Pitout, 2019; Van Duin and Paterson, 2016). Among the most alarming resistance mechanisms is the production of extended-spectrum β-lactamases (ESBLs), enzymes that inactivate a broad spectrum of β-lactam antibiotics, including cephalosporin, penicillin, and monobactam (El-Sayed et al., 2022; Superti et al., 2009). Clinically, the most significant ESBL-producing variants belong to the *CTX-M*, *TEM*, and *SHV* families. The frequent localization of *bla-ESBL* genes on plasmids, together with their close association with mobile genetic elements such as transposons and insertion sequences, has facilitated their rapid and widespread dissemination. Horizontal gene transfer further accelerates this process, enabling efficient transmission of ESBL genes to a wide variety of hosts (Ewers et al., 2021). ESBL-producing *Enterobacteriaceae*, particularly *E. coli* and *K. pneumoniae*, are currently recognized as critical priority pathogens due to their widespread occurrence and association with serious infections in humans and animals (Ribeiro et al., 2024; Ibrahim et al., 2023). The epidemiology of ESBL-producing pathogens has shifted from being exclusively confined to healthcare facilities to becoming a significant problem in the community, food chain, and environment. Food-chain animals serve as an important reservoir of ESBL-producing pathogens, with numerous studies documenting their colonization of poultry (Badr et al., 2022; Dierikx et al., 2013) and dairy cattle worldwide (Kürekci et al., 2024). These super-variants can spread widely via contamination at multiple stages of the food production chain, including on farms, during slaughter or processing, and in retail stores (Perestrelo et al., 2022). Such dynamics not only pose a threat to animal health and livestock productivity, but also represent potential sources of zoonotic disease transmission to humans, either through direct contact, environmental exposure, or consumption of contaminated animal byproducts.

Workers in close contact with animals, such as farmers, slaughterhouse staff, and food handlers, are particularly at risk of colonization with ESBL-producing pathogens (Subramanya et al., 2021); (Ribeiro et al., 2024). The occupational interface enables resistant bacteria to move between animals and humans, providing opportunities for horizontal gene transfer and persistence in human populations. Beyond the direct animal–human interactions, the surrounding environment of soil, water, and animal manure represents an additional reservoir and pathway for the spread of resistant bacteria and related genes (Wielders et al., 2020; Blaak et al., 2015). These interconnected domains illuminate the significance of adopting a One Health approach, recognizing that human, animal, and environmental health are intrinsically linked.

A significant and underexplored dimension of ESBL epidemiology is the role of seasonal variation. Environmental and management conditions change with seasons, influencing bacterial survival, shedding, and transmission (Rose et al., 2001). For example high temperatures and humidity during summer may affect bacterial survival and persistence in the environment, while seasonal changes in animal production cycles, antimicrobial use, and human–animal interactions may also affect prevalence rates. Some studies suggest that resistance levels in foodborne pathogens may peak during certain times of the year (Rose et al., 2001; Hellberg and Chu, 2016) but comprehensive data remain limited. Understanding these temporal patterns is critically important for identifying periods of high risk for disease outbreaks and designing timely intervention approaches.

Studying the seasonal influence on the prevalence of ESBL-producing bacteria among humans, animals, and the environment in Egypt is significantly important for designing targeted and cost-effective interventions. Seasonal characteristics in Egypt, such as hot summers, mild winters, and irrigated agriculture, create fluctuating temperature and humidity conditions that can affect the survival, shedding, and transmission of bacteria along the food chain from farms and live bird markets to slaughterhouses and retail outlets. Seasonal shifts in animal production cycles, disease burden, and antimicrobial use may amplify selective pressure on ESBL producers in poultry and dairy cattle, while increased contact with surface water and irrigation canals may facilitate environmental spread and human exposure.

Identifying peak transmission times and locations enables public health and veterinary services to time screening, biosafety measures, hygiene enhancements, waste and water management controls, and antimicrobial management strategies to achieve maximum impact. Adopting a seasonally- based One Health strategy in Egypt, can therefore highlight transmission hotspots at the human-animal-environment interface, improve risk assessment related to foodborne and occupational exposure, and ultimately reduce the risk of resistant infections while preserving the effectiveness of critically important antibiotics.

Therefore, the current study aims to investigate the impact of seasonal variation on the prevalence of ESBL-producing *E. coli* and *K. pneumoniae* across food-chain animals, co-workers, and the surrounding environment in Fayoum Governorate, Upper Egypt, an area around 1800 km2 characterized by high animal population density and intensive farming activities with wide varieties of livestock, mostly poultry and cattle.

## 2 Materials and methods

### 2.1 Study area

We planned to screen different samples from multiple food chain sources, including live food-producing animals (cattle and poultry), surrounding environment (feed, water, farm slurry), farm workers and hospitalized patients, from of seven districts (Markas El-Fayoum, Pander El-Fayoum, Yousef-Elsadik, Senores, Tamiya, Ebshway and Itsa) of Fayoum governorate (29.3565° N, 30.6200° E).

### 2.2 Samples collection

#### 2.2.1 Poultry samples

A total of 2,890 samples were collected from poultry farms and slaughterhouses across three seasons: summer (*n* = 1,130; 500 flocks, 630 slaughtered), fall (*n* = 860; 500 flocks, 360 slaughtered), and winter (*n* = 900; 500 flocks, 400 slaughtered). For each flock, ten cloacal swabs were pooled into a single composite sample. From each slaughterhouse, ten cecal content samples were collected and pooled together.

#### 2.2.2 Dairy cattle samples

From 16 dairy farms, milk samples were collected in summer (*n* = 22; 12 bulk tank, 10 mastitis cases), fall (*n* = 38; 26 bulk tanks, 12 mastitis cases), and winter (*n* = 28; 22 bulk tanks, 6 mastitis cases). Rectal swabs were also collected: summer (*n* = 320; 310 from apparently healthy cows, 10 from diseased cows), fall (*n* = 328; 316 from apparently healthy cows, 12 from diseased cows), and winter (*n* = 128; 122 from apparently healthy cows, 6 from diseased cows).

#### 2.2.3 Environmental samples

From poultry farms, 508 environmental samples were collected: summer (*n* = 169), fall (*n* = 170), and winter (*n* = 169). Summer samples comprised water (*n* = 55), feed (*n* = 20), litter (*n* = 50), and surfaces of knives/cutting boards (*n* = 44). Fall samples included water (*n* = 60), feed (*n* = 20), litter (*n* = 50), and knife/cutting board surfaces (*n* = 40). Winter samples included water (*n* = 70), feed (*n* = 20), litter (*n* = 40), and knife/cutting board surfaces (*n* = 39).

From dairy cattle farms, 75 environmental samples were collected: summer (*n* = 26), fall (*n* = 22), and winter (*n* = 27). Summer samples comprised water (*n* = 10), feed (*n* = 6), and slurry (*n* = 10). Fall samples included water (*n* = 10), feed (*n* = 6), and slurry (*n* = 6). Winter samples included water (*n* = 9), feed (*n* = 7), and slurry (*n* = 11).

#### 2.2.4 Human samples

Anonymized fecal specimens were obtained from bacteriological laboratories conducting routine occupational health monitoring of farm workers and also receive samples from nearby hospitals, where samples were accessed after completion of diagnostic testing and prior to disposal. No direct contact occurred between the research team and donors; all specimens were received in fully de-identified form (Supplementary Figure S1). Seasonal collections included: summer (*n* = 58 farm workers, *n* = 50 hospitalized patients), fall (*n* = 35 farm workers, *n* = 40 hospitalized patients), and winter (*n* = 25 farm workers, *n* = 40 hospitalized patients).

All samples were collected under aseptic conditions, immediately placed on ice, and transferred to a bacteriological laboratory for further analysis.

### 2.3 Isolation and biochemical identification of *Escherichia coli* and *Klebsiella pneumoniae* from the collected samples

Cloacal swabs, rectal swabs from dairy cattle, and fecal samples from hospitalized patient or from farm workers were streaked onto Eosin Methylene Blue Agar (EMBA; Himedia, Mumbai, India) plates (Badr et al., 2022; Mustika et al., 2024). Environmental samples were also obtained, including swabs from knives and cutting boards in slaughterhouses (20–40 cm^2^ surface area). These were collected using sterile cotton swabs moistened with buffered peptone water (Sebsibe and Asfaw, 2020), aseptically transferred into 10 mL sterile peptone water, and vortexed for 30 s to release microorganisms. Additionally, 10 g of cecal content, 25 g each of feed, slurry, and litter, and 25 mL each of water, tank milk, and mastitis milk samples were collected. All samples were subjected to serial decimal dilution in sterile saline solution (0.85% NaCl). Aliquots (1 mL) from appropriate dilutions were dispensed into sterile Petri dishes, overlaid with molten EMBA, gently mixed, and allowed to solidify (Sebsibe and Asfaw, 2020; International Organization for Standardization, 2013). Plates were incubated at 37 °C for 24–48 h to isolate and identify *E. coli* and *K. pneumoniae*. On EMBA media, *E. coli* colonies typically exhibit a metallic green sheen, whereas *K. pneumoniae* colonies typically appear mucoid pink. Colonies were subsequently enumerated and purified for further identification.

Presumptive *E. coli* and *K. pneumoniae* colonies were subjected to primary biochemical examination using IMViC tests (indole, methyl red, Voges-Proskauer, and citrate utilization), along with oxidase and catalase tests. In addition, identification of suspected *E. coli* and *K. pneumoniae* isolates was performed using the VITEK® 2 Compact system (bioMérieux, Marcy-l’Étoile, France) (Funke et al., 1998). Isolates were prepared as standardized suspensions in 0.45% NaCl, inoculated into GN (Gram-negative) identification cards, and incubated in the automated system. Biochemical profiles were interpreted by the VITEK® 2 software, and species-level identification was determined according to the manufacturer’s guidelines.

### 2.4 ESBL-phenotypic characterization of *Escherichia coli* and *Klebsiella pneumoniae* isolates

The biochemically/VITEK positive isolates for *E. coli* and *K. pneumoniae* were then cultured on HiCrome ESBL agar media (Himedia ®, Mumbai, India). This is a chromogenic screening medium designed for the selective isolation of ESBL-producing organisms. The bacteria that produce ESBL are distinguished by color using a chromogenic combination. ESBL producing *E. coli* grow as either pink or purple colonies whereas ESBL producing *K. pneumoniae* grow as bluish green colonies. Moreover, the phenotypic identification of ESBL-producing bacteria also obtained through double synergy test (Badr et al., 2022; Bubpamala et al., 2018) and antibiotic resistance pattern (Ryoo et al., 2005; Balta et al., 2024).

#### 2.4.1 Double disc synergy (DDST)

An amoxicillin-clavulanic disk (AMC, 30 μg) was placed 20 mm (center to center) from each antibiotic disc third-generation cephalosporins: Ceftazidime, Cefotaxime, and Ceftriaxone (30 μg each); and fourth-generation cephalosporin: Cefepime (30 μg) on Mueller-Hinton Agar plate inoculated with the test isolates (Badr et al., 2022). Following a 24-h incubation at 37 °C, an abrupt rise or augmentation of the inhibition zone of any cephalosporin discs toward to the AMC disc was deemed indicative of ESBL production.

#### 2.4.2 Antibiotic resistance pattern

Antibiotics-ESBL screening was carried out by Kirby-Bauer technique employed the disk diffusion test to produce qualitative classifications of susceptibility, including sensitive, intermediate, and resistant evaluations. This method based on various cephalosporins according to the Clinical and Laboratory Standards Institute (CLSI) standard. The antibiotics concentrations were selected according to CLSI guidelines. Isolates with an inhibition zone size of ≤22 mm with ceftazidime (30 μg), ≤25 mm with ceftriaxone (30 μg), ≤27 mm with cefotaxime (30 μg), ≤18 mm Cefepime (30 μg), and ≤15 mm with aztreonam (10 μg) were identified as potential ESBL producers CLSI (Clinical and Laboratory Standards Institute) (2021) Performance standards for antimicrobial susceptibility testing. 25th informational supplement, Clinical and Laboratory Standards Institute, Wayne, M100-S25. CLSI (2023). Performance Standards for Antimicrobial Susceptibility Testing. 33rd ed. CLSI supplement M100. Furthermore, antibiotic susceptibility profiling was performed using the Kirby–Bauer disk diffusion method (Kirby and Bauer, 1966) against eight antibiotics (Bioanalyse, Ankara, Turkey): oxytetracycline (TE, 30 µg), colistin (CT, 10 µg), meropenem (MEM, 10 µg), ampicillin (AM, 10 µg), ciprofloxacin (CIP, 5 µg), chloramphenicol (C, 30 µg), amoxicillin/clavulanate (AMC, 20/10 µg), and sulfamethoxazole/trimethoprim (SXT, 1.25/23.75 µg). The results of antibiotic susceptibility testing were interpreted according to the guidelines of the Clinical and Laboratory Standards Institute (CLSI) (Balta et al., 2024).

### 2.5 ESBL-genotypic characterization of *Escherichia coli* and *Klebsiella pneumoniae* isolates

The phenotypically ESBL-positive *E. coli* and *K. pneumoniae* isolates were examined by multiplex PCR to detect the presence of ESBL genes, including *blaTEM*, *blaSHV*, and *blaCTX-M* (groups 1 and 9) as previously described (Bubpamala et al., 2018; Ryoo et al., 2005). Briefly, DNA was extracted from the phenotypically ESBL-positive isolates using the GeneDireX DNA Extraction Kit (Taiwan) following the manufacturer’s instructions. The extracted DNA was then amplified using gene-specific multiplex PCR reactions. The cycling conditions were as follows: initial denaturation at 94 °C for 10 min, followed by 30 cycles of denaturation at 94 °C for 30 s, annealing at 60.1 °C for 35 s, and extension at 72 °C for 1 min. A final extension step was performed at 72 °C for 9 min. The primers sequences used in this study are listed in Table 1.

**Table 1:** Target genes and their primers.

Target gene	Forward primer (5′–3′)	Reverse primer (5′–3′)	Amplicon size (bp)
*blaTEM*	CATTTCCGTGTCGCCCTTATTC	CGTTCATCCATAGTTGCCTGAC	800
*blaSHV*	AGCCGCTTGAGCAAATTAAAC	ATCCCGCAGATAAATCACCAC	713
*blaCTX-M1*	TTAGGAAATGTGCCGCTGTA	CGATATCGTTGGTGGTACCAT	878
*blaCTX-M9*	GCA GAT AAT ACG CAG GTG	CGG CGT GGT GGT GTC TCT	164

Additionally, in order to study the distribution of ESBL genes among bacterial isolates was analyzed in relation to their antibiotic resistance profiles using hierarchical heat-map visualization.

### 2.6 Correlation analysis of ESBL genes and antibiotic resistance profiles

Pairwise correlations between antibiotic resistance genes and antimicrobial susceptibility profiles were calculated using the Pearson correlation coefficient (r). Only numeric variables were included, with categorical data converted to ordinal values (0 = sensitive/absent, 1 = intermediate, 2 = resistant/present). Zero-variance variables were excluded to avoid bias. Correlation matrices were computed using the cor() function with pairwise complete observations. Corresponding *p*-values were obtained using the cor() function and subsequently adjusted for multiple comparisons using the Benjamini–Hochberg false discovery rate (FDR) method. Only correlations that remained significant after FDR correction were interpreted.

### 2.7 Statistical analysis

Statistical analysis of the data was conducted using SPSS version 22 via Chi-Square tests and Fisher’s Exact Test. The Chi-Square test is used to determine the association between samples sources, seasons and isolation rate for *E. coli* and *K. pneumoniae* and ESBL-production. The result of *E. coli* and *K. pneumoniae* in different demographic variables by Chi-Square test and for cells with expected count below 5 were tested using Fisher’s exact test and all statistical analysis were performed at *p* < 0.05. Statistical significance of correlations was assessed using cor.test, and corresponding *p*-values were adjusted at confidence level 95%. Pearson correlation coefficient (r) and all data was showed and plotted using R version 4.3.2 Software.

## 3 Results

### 3.1 Prevalence rate of *Escherichia coli* and *Klebsiella pneumoniae* isolated from the collected samples across three seasons

During summer season, a total of 500 cloacal swabs and 630 cecal cores were collected from poultry and pooled into 50 and 63 samples, respectively (Table 2). From these, 14 *E. coli* and 2 *K. pneumoniae* isolates were recovered from cloacal swabs, while 11 *E. coli* and 6 *K. pneumoniae* were obtained from cecal cores. Poultry farm environmental samples (*n* = 169; water, feed, litter, and surface swabs), yielded 36 *E. coli* and 14 *K. pneumoniae* isolates. Additionally, samples from poultry farm workers (*n* = 51) recovered 26 *E. coli* and 7 *K. pneumoniae*. In dairy farms, rectal swabs from healthy cows (*n* = 320) yielded 34 *E. coli* and 8 *K. pneumoniae*, while diseased cows (*n* = 10) produced 4 *E. coli* and 2 *K. pneumoniae*. Mastitic milk samples (*n* = 10) yielded 7 *E. coli* and 2 *K. pneumoniae*, whereas bulk tank milk (*n* = 12) produced 2 *E. coli* only. Environmental samples (*n* = 26) recovered 2 *E. coli* and 2 *K. pneumoniae*, but no isolates were obtained from dairy workers (*n* = 7). From hospitalized patients, fecal samples (*n* = 50) yielded 9 *E. coli* and 1 *K. pneumoniae*.

**Table 2:** Prevalence rate of *Escherichia coli* and *Klebsiella pneumoniae* in the summer season based on VITEK.

Sample source		Poultry farms							Dairy farms								Hospitals
Sample type		Cloacal swabs	Cecal core	Environment (n:169)				Farm Workers	Rectal swabs n:320		Milk n:22		Environment n:26			Farm workers	Fecal swabs
		Live	Slaughtered	Water	Feed	Litter	Surface	Fecal swab	Healthy	Diseased	Mastitic	Normal	Water	Feed	Slurry	Fecal samples	
Collected No.		50	63	55	20	50	44	51	310	10	10	12	10	6	10	7	50
Bacterial species	*Escherichia coli N* (%)	14 (28)	11(17.46)	10 (18.18)	5 (25)	12 (24)	9 (20.45)	26 (50.98)	34 (10.96)	4 (40)	7 (70)	2 (16.66)	1 (10)	0	1 (10)	0	9 (14)
	*Klebsiella pneumoniae N* (%)	2 (4)	6 (9.5)	9 (16.36)	1 (5)	2 (4)	2 (4.5)	7 (13.72)	8 (2.58)	2 (20)	2 (20)	0	0	2 (33.3)	0	0	1 (2)
*P* value		0.001*	0.192	0.001*				0.000*	0.000*	0.628	0.070	0.478	1			a	0.008*

*Statistically significant at *P* value <0.05.

a. No statistics are computed because results is a constant.

In the fall season, cloacal swabs (*n* = 50 pools) yielded 8 *E. coli* and 3 *K. pneumoniae*, while cecal cores (*n* = 63 pools) produced 9 *E. coli* with no *K. pneumoniae*. Poultry environmental samples (*n* = 170) recovered 11 *E. coli* only, and farm workers (*n* = 25) yielded 19 *E. coli* with no *K. pneumoniae*. In dairy farms, rectal swabs from healthy cows (*n* = 316) yielded 57 *E. coli* and 1 *K. pneumoniae*, while diseased cows (*n* = 12) produced 2 *E. coli* only. Dairy environmental samples (*n* = 22) yielded 7 *E. coli*. From hospitalized patients, fecal samples (*n* = 40) yielded 5 *E. coli* and 1 *K. pneumoniae* (Table 3).

**Table 3:** Prevalence rate of *Escherichia coli* and *Klebsiella pneumoniae* in the fall season based on VITEK.

Sample source		Poultry farms							Dairy farms								Hospitals
Sample type		Cloacal swabs	Cecal core	Environment (n:170)				Farm workers	Rectal swabs n:328		Milk n:38		Environment n:22			Farm workers	Fecal samples
		Live	Slaughtered	Water	Feed	Litter	Surface	Fecal samples	Healthy	Diseased	Mastitic	Normal	Water	Feed	Slurry	Fecal samples	
Collected No.		50	36	60	20	50	40	25	316	12	12	26	10	6	6	10	40
Bacterial species	*Escherichia coli N* (%)	8 (16)	9 (25)	2 (3.33)	0	9 (18)	0	19 (76)	57 (18.03)	2 (16.66)	2 (16.66)	0	0	0	7 (116)	0	5 (12.5)
	*Klebsiella pneumoniae N* (%)	3 (6)	0	0	0	0	0	0	1 (0.31)	0	0	2 (7.69)	0	0	0	0	1 (2.5)
*P* value		0.110*	0.002*	0.001*				0.000*	0.000*	0.478	0.478	0.490	0.009*			a	0.201

*Statistically significant at *P* value <0.05.

a. No statistics are computed because results is a constant.

In the winter season, cloacal swabs (*n* = 50) yielded 2 *E. coli* and 1 *K. pneumoniae*, while pooled cecal samples (*n* = 40) yielded 8 *E. coli* and 3 *K. pneumoniae*. Poultry farm environmental samples (*n* = 169) recovered 36 *E. coli* and 12 *K. pneumoniae*, whereas no isolates were obtained from poultry worker fecal samples (*n* = 10). In dairy farms, rectal swabs from healthy cows (*n* = 122) yielded 15 *E. coli* and 4 *K. pneumoniae*, while samples from diseased cows (*n* = 6) were negative for both organisms. Neither mastitic milk (*n* = 6) nor normal tank milk (*n* = 22) yielded any isolates. Environmental samples (*n* = 27) recovered 3 *E. coli* and 1 *K. pneumoniae*, whereas dairy farm workers (*n* = 15) yielded 13 *E. coli* but no *K. pneumoniae*. Similarly, fecal samples of hospitalized patients (*n* = 40) recovered only 16 *E. coli* (Table 4).

**Table 4:** Prevalence rate of *Escherichia coli* and *Klebsiella pneumoniae* in the winter season based on VITEK.

Source		Poultry farms							Dairy farms								Hospitals
Sample type		Cloacal swabs	Cecal core	Environment (n:169)				Farm Workers	Rectal swabs n:128		Milk n:28		Environment n:27			Farm workers	Fecal swabs
		Live	Slaughtered	Water	Feed	Litter	Surface	Fecal swab	Healthy	Diseased	Mastitic	Normal	Water	Feed	Slurry	Fecal samples	
Collected No.		50	40	70	20	40	39	10	122	6	6	22	9	7	11	15	40
Bacterial species	*Escherichia coli N* (%)	2 (4)	8 (20)	11 (15.71)	1 (5)	22 (55)	2 (5.12)	0	15 (12.29)	0	0	0	1 (11.11)	2 (28.57)	0	13 (86.66)	16 (40)
	*Klebsiella pneumoniae N* (%)	1 (2)	3 (7.5)	1 (1.42)	4 (20)	3 (7.5)	0	0	4 (3.27)	0	0	0	1 (11.11)	0	0	0	0
*P* value		1.000	0.105	0.000*				a	0.009*	a	a	a	0.610			0.000*	0.000*

*Statistically significant at *P* value <0.05.

a. No statistics are computed because results is a constant.

**Figure 1:**
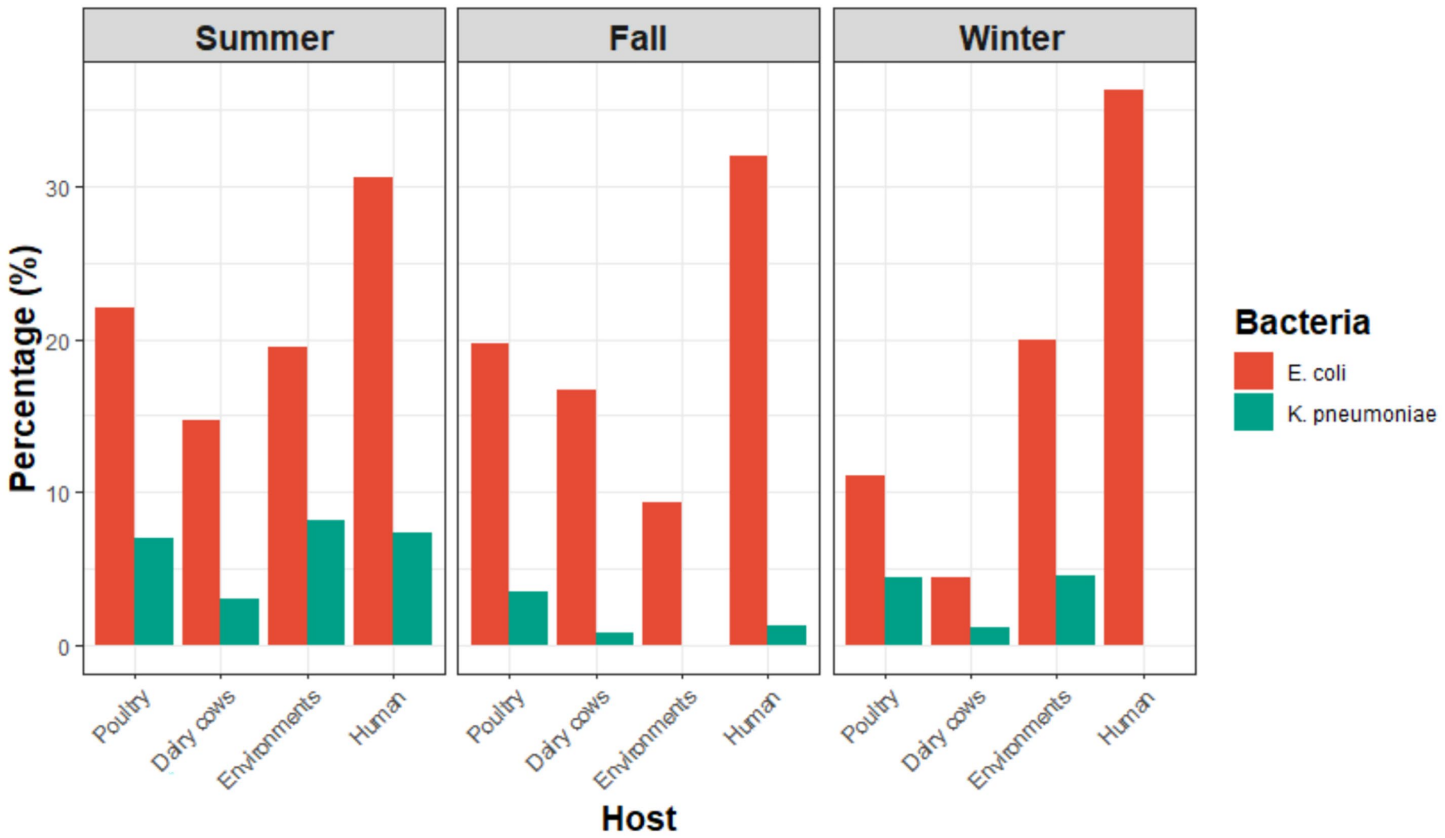
The percentage of *Escherichia coli* and *Klebsiella pneumoniae* isolates revered from different hosts (Poultry, dairy cows, environments, co-workers and hospitalized patients) across three seasons: Summer, fall, and winter. Data show variations across sources and seasons, with significant seasonal effects.

As shown in Figure 1 and Supplementary Table S1, *E. coli* prevalence in poultry samples ranged from 22.1% in summer to 19.8% in fall and 11.1% in winter, while *K. pneumoniae* varied between 7.07% and 3.48–4.44%, with no significant seasonal effect observed. In dairy cows, *E. coli* prevalence decreased sharply from 14.68–16.7% in summer and fall to 4.4% in winter, and *K. pneumoniae* dropped from 3.1% to below 1.2%, showing significant seasonal variation with winter as the lowest-prevalence season (*p* = 0.043). Environmental samples showed fluctuating trends, with *E. coli* peaking in summer (19.5%) and winter (19.9%) but dropping in fall (9.4%), while *K. pneumoniae* was present in summer (8.2%) and winter (4.6%) but absent in fall, both demonstrating significant seasonal effects (*E. coli p* = 0.007 and *K. pneumoniae p* = 0.000). Among farm workers, *E. coli* prevalence remained consistently high across seasons (44.8–54.3%) with no significant variation, while *K. pneumoniae* was detected only in summer (12.1%), reflecting significant seasonality (*p* = 0.021). In hospitalized patients, *E. coli* prevalence ranged from 12.5 to 29.1%, and *K. pneumoniae* remained very low (2–2.5%), with no significant seasonal differences. When comparing across sources within the same season, *E. coli* showed highly significant differences in all seasons (p = 0.000), while *K. pneumoniae* differed significantly in summer (*p* = 0.016) and winter (*p* = 0.045) but not fall.

### 3.2 ESBL- identification

#### 3.2.1 Phenotypic detection of ESBL-producing isolates across seasons

The *E. coli* and *K. pneumoniae* isolates recovered from different hosts across the three seasons were subjected to phenotypic characterization using HiCrome ESBL agar. HiCrome-positive isolates were subsequently confirmed by the ESBL screening antibiotic test and the double-disk synergy test (DDST). The presence of either resistance to at least one third- or fourth-generation cephalosporin, or a synergistic effect of amoxicillin-clavulanic with any cephalosporin antibiotic, was considered phenotypically positive, as detailed in Tables 5–7.

**Table 5:** ESBL-phenotypic characterization of HiCrome ESBL- positive isolates recovered in summer season.

Source	Bacterial strains (n)	ESBL- phenotypes detection using disk diffusion method															
		ESBL-screening antibiotics (Resistance %)															DDST Positive (n)
		3rd generation cephalosporins									4th generation cephalosporin			Monobactam			
		Ceftazidime (CAZ)			Ceftriaxone (CRO)			Cefotaxime (CTX)			Cefepime (CEF)			Aztreonam (ATM)			
		R	I	S	R	I	S	R	I	S	R	I	S	R	I	S	
Poultry	*Escherichia coli* (13)	13 (100)	0	0	9 (69)	3 (23)	1 (7.6)	13 (100)	0	0	12 (92.3)	1 (7.6)	0	9 (69.2)	4 (30.7)	0	4 (30.7)
	*Klebsiella pneumoniae* (5)	4 (80)	1 (20)	0	5 (100)	0	0	5 (100)	0	0	5 (100)	0	0	5 (100)	0	0	0
Dairy cattle	*Escherichia coli* (5)	2 (40)	3 (60)	0	1 (20)	3 (60)	1 (20)	4 (80)	0	1 (20)	1 (20)	3 (60)	1 (20)	3 (60)	1 (20)	1 (20)	4 (80)
	*Klebsiella pneumoniae* (1)	(100)	0	0	1 (100)	0	0	1 (100)	0	0	1 (100)	0	0	1 (100)	0	0	1 (100)
Environment	*Escherichia coli* (18)	13 (72)	4 (22.2)	1 (5.5)	12 (66.6)	4 (22.2)	2 (11.1)	17 (94.4)	0	1 (5.5)	16 (88.8)	1 (5.5)	1 (5.5)	8 (44.4)	8 (44.4)	2 (11.1)	4 (22.2)
	*Klebsiella pneumoniae* (12)	12 (100)	0	0	11 (91.6)	1 (8.3)	0	(12)	0	0	11 (91.6)	1 (8.3)	0	10 (83)	2 (16.6)	0	1 (8.3)
Human	*Escherichia coli* (16)	13 (81.25)	3 (18.75)	0	9 (56)	6 (37.5)	1 (6.25)	16 (100)	0	0	12 (75)	2 (12.5)	2 (12.5)	9 (56)	6 (37.5)	1 (6)	9 (56.25)
	*Klebsiella pneumoniae* (5)	4 (80)	1 (20)	0	3 (60)	2 (40)	0	5 (100)	0	0	5 (100)	0	0	4 (80)	0	1 (20)	3 (60)

**Table 6:** ESBL-phenotypic characterization of HiCrome ESBL- positive isolates recovered in fall season.

Source	Bacterial strains (n)	ESBL- phenotypes detection using disk diffusion method															
		ESBL-screening antibiotics (Resistance %)															DDST Positive (n)
		3rd generation cephalosporins									4th generation cephalosporin			Monobactam			
		Ceftazidime (CAZ)			Ceftriaxone (CRO)			Cefotaxime (CTX)			Cefepime (CEF)			Aztreonam (ATM)			
		R	I	S	R	I	S	R	I	S	R	I	S	R	I	S	
Poultry	*Escherichia coli* (9)	7 (77)	2 (22)	0	7 (77)	1 (11)	1 (11)	9 (100)	0	0	7 (77)	1 (11)	1 (11)	7 (77)	2 (22)	0	2 (22)
	*Klebsiella pneumoniae* (0)	0	0	0	0	0	0	0	0	0	0	0	0	0	0	0	0
Dairy cattle	*Escherichia coli* (17)	8 (47)	8 (47)	1 (5.88)	8 (47)	5	4 (23)	15 (88.2)	0	2 (11)	8 (47)	5 (29)	4 (23)	12 (70.6)	4 (23)	1 (5)	8 (47)
	*Klebsiella pneumoniae* (1)	0	1 (100)	0	1 (100)	0	0	1 (100)	0	0	0	1 (100)	0	0	1 (100)	0	0
Environmental	*Escherichia coli* (3)	3 (100)	0	0	3 (100)	0	0	3 (100)	0	0	2 (66)	1 (33)	0	3 (100)	0	0	1 (33)
	*Klebsiella pneumoniae* (0)	0	0	0	0	0	0	0	0	0	0	0	0	0	0	0	0
Human	*E.coli* (15)	13 (86)	2 (13)	0	13 (86)	2 (13)	0	14 (93.3)	0	1 (6.6)	11 (73)	4 (26.6)	0	13 (86)	2 (13)	0	7 (46.6)
	*Klebsiella pneumoniae* (0)	0	0	0	0	0	0	0	0	0	0	0	0	0	0	0	0

**Table 7:** ESBL-phenotypic characterization of HiCrome ESBL- positive isolates recovered in winter season.

Source	Bacterial strains (n)	ESBL- phenotypes detection using disk diffusion method															
		ESBL-screening antibiotics (Resistance %)															DDST positive (n)
		3rd generation cephalosporins									4th generation cephalosporin			Monobactam			
		Ceftazidime (CAZ)			Ceftriaxone (CRO)			Cefotaxime (CTX)			Cefepime (CEF)			Aztreonam (ATM)			
		R	I	S	R	I	S	R	I	S	R	I	S	R	I	S	
Poultry	*Escherichia coli* (5)	4 (80)	1 (20)	0	5 (100)	0	0	4 (80)	0	1 (20)	5 (100)	0	0	4 (80)	0	1 (20)	2 (40)
	*Klebsiella pneumoniae* (3)	2 (66.6)	1 (33.3)	0	2 (66.6)	0	1 (33.3)	3,100	0	0	3,100	0	0	2 (66.6)	0	1 33.3	2 (66.6)
Dairy cattle	*Escherichia coli* (2)	0	2 (100)	0	1 (50)	0	1 (50)	2 (100)	0	0	2 (100)	0	0	2 (100)	0	0	0
	*Klebsiella pneumoniae* (1)	1 (100)	0	0	1 (100)	0	0	1 (100)	0	0	1 (100)	0	0	1 (100)	0	0	0
Environment	*E.coli* (9)	8 (88)	1 (12.5)	0	8 (88)	0	1 (12.5)	8 (88)	0	1 (12.5)	6 (66.6)	0	3 (33)	6 (66.6)	0	3 (33)	3 (33)
	*Klebsiella pneumoniae* (4)	1 (25)	0	3 (75)	2 (50)	0	2 (50)	3 (75)	0	1 (25)	0	1 (25)	3 (75)	0	1 (25)	3 (75)	3 (75)
Human	*Escherichia coli* (4)	3 (75)	1 (25)	0	3 (75)	1 (25)	0	4 (100)	0	0	3 (75)	1 (25)	0	3 (75)	1 (25)	0	2 (50)
	*Klebsiella pneumoniae* (0)	0	0	0	0	0	0	0	0	0	0	0	0	0	0	0	0

Table 5 summarizes the phenotypically confirmed ESBL producers among the summer isolates. Of the 13 *E. coli* isolates recovered from poultry, 4 were confirmed as ESBL producers, whereas all *K. pneumoniae* isolates from poultry tested negative. In dairy cattle, 4 of 5 *E. coli* isolates and the single *K. pneumoniae* isolate were ESBL positive. Among environmental isolates, 4 of 18 *E. coli* and 1 of 12 *K. pneumoniae* demonstrated ESBL activity. From human samples, 9 of 16 *E. coli* isolates and 3 of 5 *K. pneumoniae* isolates were confirmed as ESBL producers.

In total, 21 of 52 *E. coli* isolates and 5 of 23 *K. pneumoniae* isolates recovered during summer were phenotypically confirmed as ESBL producers.

In the fall season, 2 of 9 *E. coli* isolates from poultry, 8 of 17 *E. coli* isolates from dairy cattle, 1 of 3 *E. coli* isolates from environmental samples, and 7 of 15 *E. coli* isolates from humans were confirmed as ESBL producers. Overall, 18 of 44 *E. coli* isolates were ESBL producers. In contrast, none of the *K. pneumoniae* isolates recovered from any host exhibited ESBL activity during fall (Table 6).

In the winter season, 2 of 5 *E. coli* and 2 of 3 *K. pneumoniae* from poultry were confirmed as ESBL producers. Neither the 2 *E. coli* nor the single *K. pneumoniae* recovered from dairy cattle tested positive. Among environmental isolates, 3 of 9 *E. coli* and 3 of 4 *K. pneumoniae* were ESBL positive, while from human samples 2 of 4 *E. coli* were positive and no *K. pneumoniae* were recovered. Overall, 7 of 20 *E. coli* and 5 of 8 *K. pneumoniae* obtained in winter were confirmed as phenotypic ESBL producers as shown in Table 7.

#### 3.2.2 Genotypic identification and seasonal distribution of ESBL-producers

**Figure 2:**
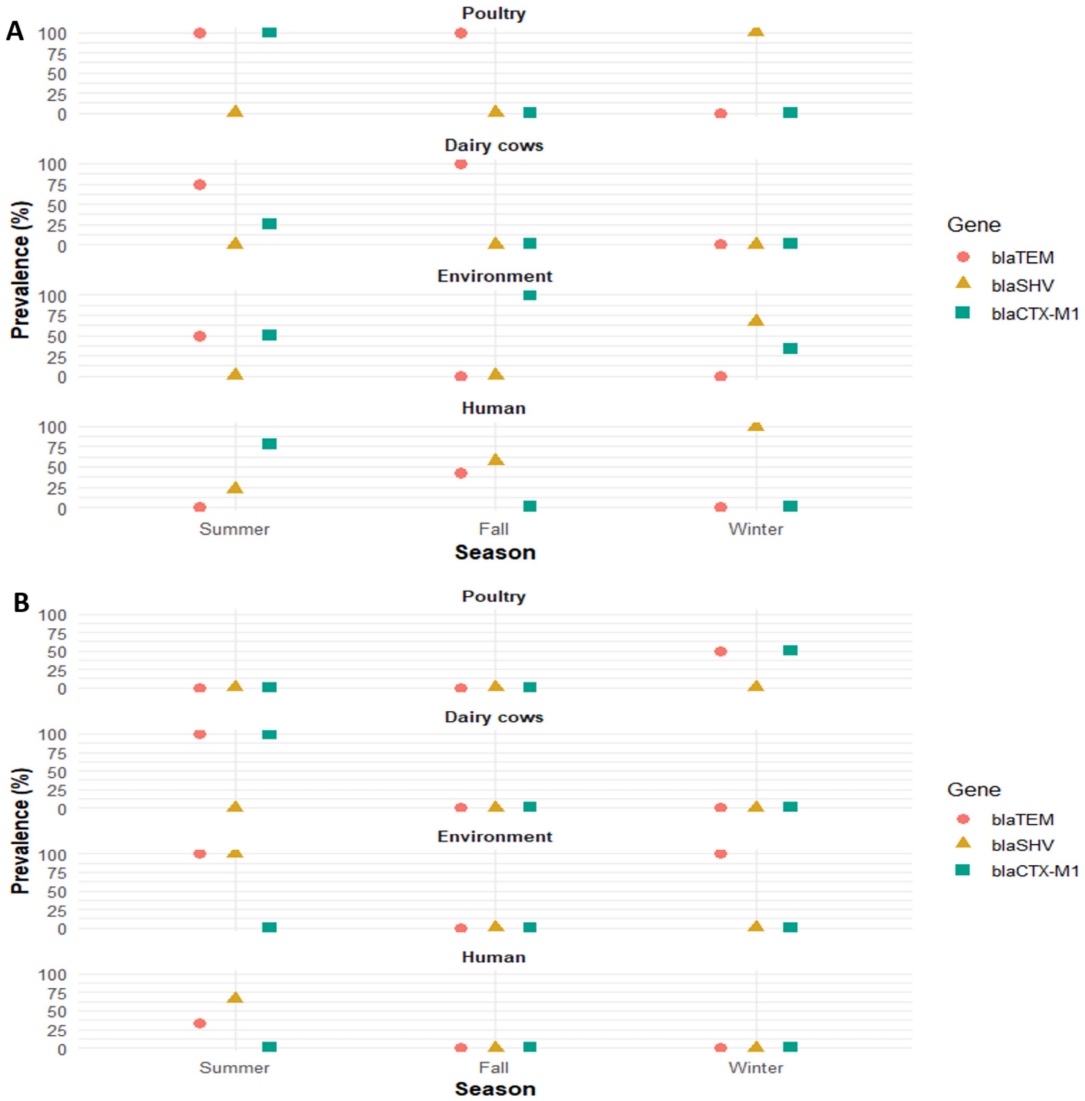
Genotypic identification and seasonal distribution of ESBL genes (*blaTEM. blaSHV, blaCTX-M1*) in *Escherichia coli* **(A)** and *Klebsiella pneumoniae* **(B)** isolated from poultry, dairy cows, environment, and human.

The PCR analysis revealed variable distribution of ESBL genes among isolates of different sources across seasons (Figure 2). In summer isolates, the *blaTEM* gene was widely detected in *E. coli* isolates from poultry (100%), dairy (75%), and environment (50%), but was absent in human isolates. In *K. pneumoniae*, *blaTEM* was found in isolates from dairy (100%), environment (100%), and some human samples (33.3%). The *blaSHV* gene was absent in *E. coli* from animal and environmental sources but was present in human isolates (22.2%). In *K. pneumoniae*, *blaSHV* was detected in environmental isolates (100%) and in humans (66.6%). The *blaCTX-M* group 1 gene showed high prevalence in *E. coli* from poultry (100%) and humans (77.7%) and was also present in dairy (25%) and environment (50%). In *K. pneumoniae*, it was detected only in dairy isolates (100%). None of the examined *E. coli* or *K. pneumoniae* isolates harbored *blaCTX-M* group 9. Among the fall isolates, the most prevalent ESBL gene was *blaTEM*, detected in 100% of *E. coli* isolates from both poultry and dairy sources. In human isolates, *blaSHV* was the most frequent (57.1%), whereas *blaTEM* was present in 42.85%. In contrast, environmental *E. coli* isolates predominantly harbored *blaCTX-M*. While the winter isolates, the *blaTEM* gene was absent in *E. coli* from all examined sources but was detected in *K. pneumoniae* isolates from poultry (50%) and from all environmental samples (100%). The *blaSHV* gene was consistently present in *E. coli* isolates from poultry (100%), environment (66.6%), and humans (100%), whereas it was not detected in *K. pneumoniae*. The *blaCTX-M* group 1 gene was detected in *E. coli* isolates from the environment (33.3%) and in *K. pneumoniae* isolates from poultry (50%). However, *blaCTX-M* group 1 gene was absent in dairy and human isolates. None of the *E. coli* or *K. pneumoniae* isolates harbored *blaCTX-M* group 9.

### 3.3 Prevalence of ESBL-producing *Escherichia coli* and *Klebsiella pneumoniae* across seasons

**Figure 3:**
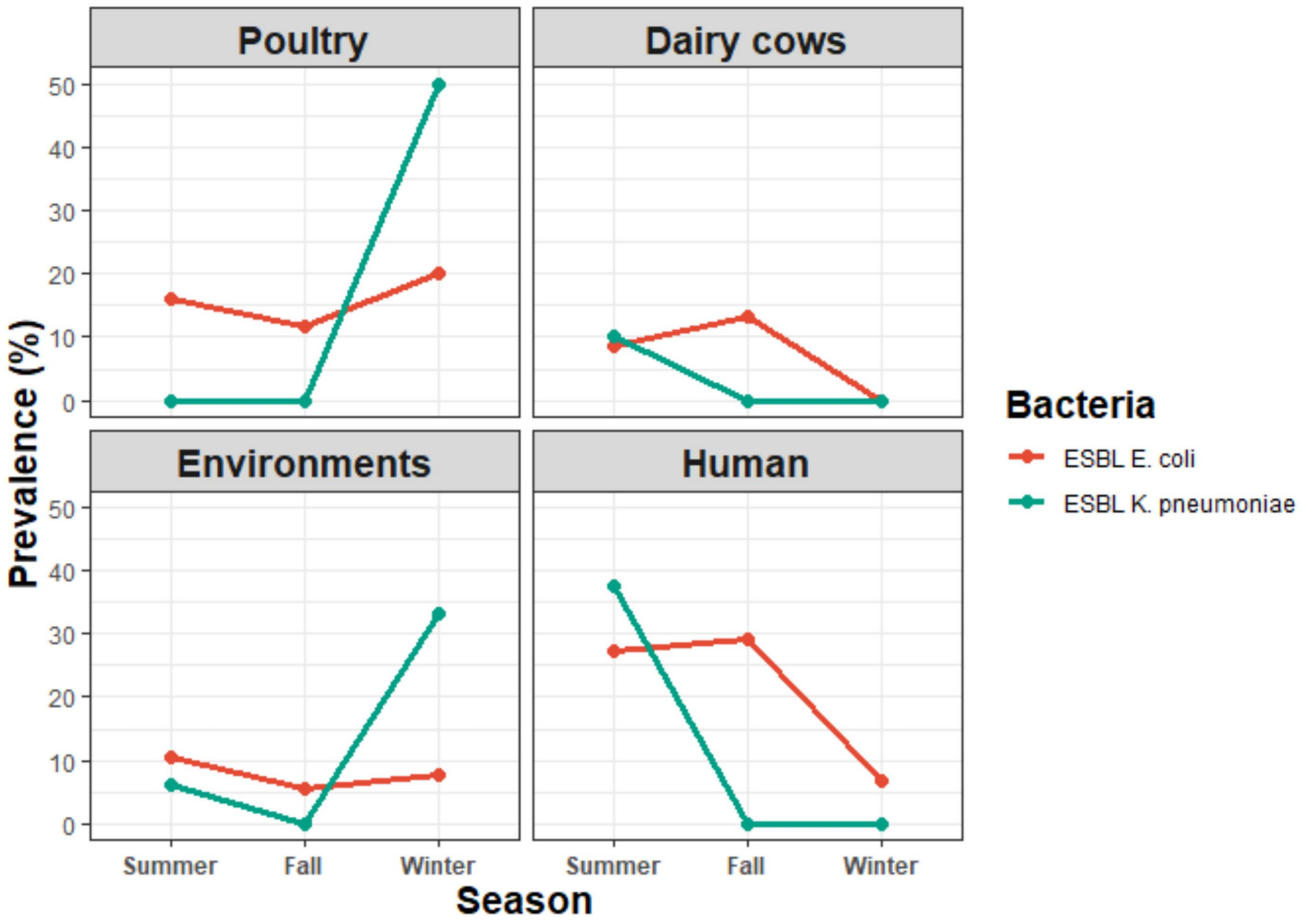
Seasonal prevalence of phenotypically and genotypically ESBL-producing *Escherichia coli* and *Klebsiella pneumoniae* in poultry, dairy cows, environment, and human. Statistical significance was determined at *p* < 0.05.

As shown in Figure 3 and Supplementary Table S2, ESBL-producing *E. coli* from poultry was detected at comparable rates in summer (16%) and fall (11.76%), with a slight increase in winter (20%). In contrast, ESBL-producing *K. pneumoniae* from poultry showed significant seasonal variation (*p* = 0.042), being detected only in winter (50%), but absent in summer and fall. Among dairy cow isolates, *E. coli* displayed moderate ESBL prevalence in summer (8.5%) and fall (13.11%), but none were detected in winter, while ESBL-*K. pneumoniae* (10%) was detected only in summer. Environmental *E. coli* isolates showed fluctuating ESBL-detection rates in summer (10.5%), Fall (5.5%) and winter (7.7%), while *K. pneumoniae* showed high ESBL prevalence in winter (33.3%) compared to summer (6.25%) and but absent in fall. Farm worker isolates demonstrated seasonal differences, with ESBL-producing *E. coli* identified in summer (23.08%) and fall (21.0%) but absent in winter. Human fecal samples from hospitalized patients also showed variation, with *E. coli* ESBL prevalence of 42.8% in summer, rising to 60% in fall, but decreasing to 12.5% in winter. Overall, ESBLs production in *E. coli* was comparable in summer (14.68%) and fall (15%) before declining in winter (7.5%), while in *K. pneumoniae*, the highest prevalence was in winter (29.4%) compared to lower detection in summer (11.9%) and complete absence in fall. Statistical analysis indicated significant seasonal differences in *E. coli* prevalence between sources in the fall (*p* = 0.039), whereas *K. pneumoniae* variation was significant in poultry across seasons (*p* = 0.042).

### 3.4 Antibiotic resistance-pattern of ESBL-producing *Escherichia coli* and *Klebsiella pneumoniae*

**Figure 4:**
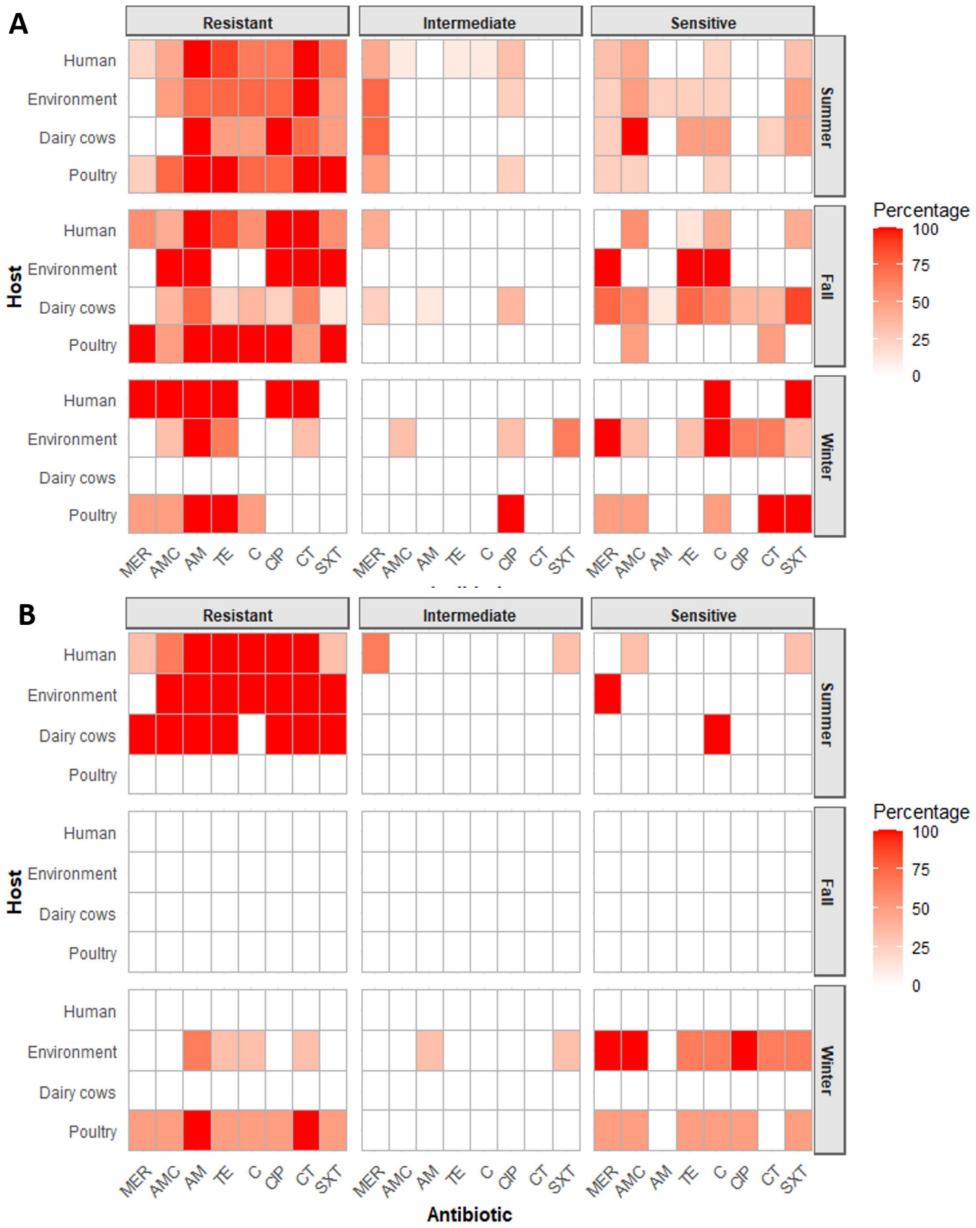
Antibiotic resistance patterns of **(A)** ESBL-*Escherichia coli* and **(B)** ESBL-*Klebsiella pneumoniae* across hosts and seasons against eight different antibiotics: oxytetracycline (TE), Colistin (CT), meropenem (MEM), ampicillin (AM), ciprofloxacin (CIP), chloramphenicol **(C)**, amoxicillin/clavulanate (AMC), and sulfamethoxazole/trimethoprim (SXT).

All the ESBL-isolates from poultry, dairy cows, environment and human were tested against 8 antibiotics including TE, CT, MEM, AM, CIP, C, AMC, and SXT. The result of *E. coli* isolates across seasons highlighted the highest and most consistent resistance with AM (100%) in poultry and human isolates during summer and fall, persisting in winter with poultry. CT resistance was also very high in summer, reaching 100% in humans, environmental, and poultry isolates. In fall, resistance remained at 100% in both human and environmental isolates, but declined in winter to 33% in environmental isolates. MEM resistance increased in fall (57.1% in humans and 100% in poultry) but declined in winter (50% in poultry and absent in the environment). CIP resistance peaked in fall (100%) among human, poultry and environment but ranged from 66.6% in human to 100% in dairy during summer but dropped to 0% in winter human isolates. STX resistance was moderate to high in summer (50–100%) and ranged from low to high (12.5–100%) in fall but declined in winter, with environmental isolates fully susceptible. Overall, resistance levels were greatest in fall, especially in human and poultry isolates, whereas winter isolates, particularly from the environment, displayed comparatively lower resistance (Figure 4A and Supplementary Tables S3, S5).

Antibiotic resistance profile of ESBL- producing *K. pneumoniae* from dairy and environment displayed complete resistance to most antibiotics in summer, with the dairy isolate resistant to all agents except C, while the environmental isolates were susceptible only to MEM. Human isolates of summer exhibited 100% resistance to AM, C, TE, CT and CIP, with lower resistance to MEM (33.3%), AMC (66.6%), and SXT (33.3%). In winter, Poultry isolates demonstrated high resistance (50–100%) to most agents. Environmental isolates showed resistance ranged from 33.3 to 66.6%, where resistance was absent (0%) in MEM, AMC, CIP, and SXT Overall, although both summer and winter isolates displayed extensive resistance, winter isolates particularly from environment showed relatively greater susceptibility compared to the uniformly resistant summer isolates (Figure 4B and Supplementary Tables S6, S7).

### 3.5 Distribution of ESBL-genes among isolates in relation to antibiotic profiling

**Figure 5:**
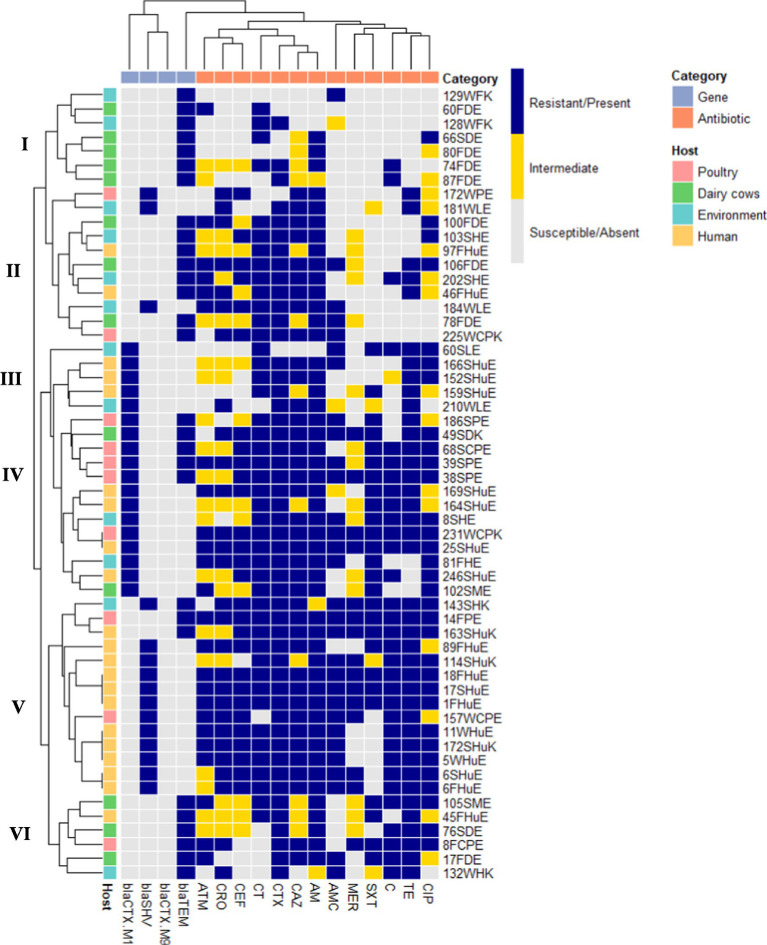
Distribution of ESBL genes (*blaTEM, blaSHV, blaCTX-M1, blaCTX-M9*) among 56 ESBL-producing *Escherichia coli* (Mendonça et al., 2007) and *Klebsiella pneumoniae* (Badr et al., 2022) in relation to antibiotic resistance profiles. Hierarchical clustering separated the isolates into six major clusters (I: VI), reflecting distinct host-associated resistance and gene distribution patterns.

A total of 56 ESBL-producing isolates of *E. coli* and *K. pneumoniae* were screened for the presence of four β-lactamase genes (*blaTEM, blaSHV, blaCTX-M1,* and *blaCTX-M9*) and thirteen antibiotic resistance phenotypes (CAZ, CRO, CTX, CEF, ATM, MER, AMC, AM, TE, C, CIP, CT, and SXT). The hierarchical clustering heatmap (Figure 5) revealed distinct distribution patterns of ESBL genes in relation to resistance against a panel of antibiotics.

Overall, the majority of isolates harbored at least one ESBL gene, with *blaTEM* (51.8%) being the most frequently detected, followed by *blaCTX-M1* (32.1%), whereas *blaSHV* (26.8%) is less common and there is no *blaCTX-M9* detected. A considerable proportion of isolates carried multiple ESBL genes simultaneously.

Phenotypically, the majority of isolates were resistant to third-generation cephalosporins, with the highest resistance against CTX (89.3%) and CAZ (69.6%). Very high resistance rates were also observed for AM (87.5%), TE (71.4%), and CT (80.3). Resistance to CIP and C was moderate (both 57.1%) followed by AMC (53.5%), SXT (46.4%), and ATM (42.8%). In contrast, carbapenem resistance (meropenem) was the lowest (26.8%), indicating that carbapenems remain the most effective antibiotics against these isolates.

Hierarchical clustering divided the 56 ESBL-producing *E. coli* and *K. pneumoniae* isolates into six distinct clusters, with clear sub-structuring by host origin, gene carriage, and antibiotic resistance profiles. Cluster I, dominated by dairy cow isolates (*n* = 5) along with two environmental isolates, showed the lowest resistance levels and primarily carried *blaTEM*. Cluster II consisted mainly of environmental isolates (*n* = 4) and mixed-source isolates (*n* = 7; including three from dairy cows, and two each from human and poultry). This cluster displayed heterogeneous but generally moderate resistance phenotypes, with the majority harboring *blaTEM*, while only three strains carried *blaSHV*. Cluster III, associated with human (*n* = 3) and environmental (*n* = 2) isolates, exhibited the highest levels of multidrug resistance and frequent co-carriage of *blaCTX-M1*. Cluster IV comprised mainly poultry (*n* = 5) and human (*n* = 4) isolates, with additional contributions from dairy cows (*n* = 2) and the environment (*n* = 2). This cluster showed high resistance profiles and was predominantly associated with *blaCTX*. Cluster V, dominated by human isolates (*n* = 11) along with two poultry and one environmental isolate, demonstrated high resistance levels, with most carrying *blaSHV* and only three harboring *blaTEM*. Finally, Cluster VI, composed mainly of dairy cow isolates (*n* = 3) and one isolate each from human, poultry, and environment, displayed generally moderate resistance profiles, with all members carrying *blaTEM*. These findings underscore the host- and environment-specific dynamics of ESBL gene dissemination, with clinical isolates serving as primary reservoirs of multidrug resistance, while animal and environmental isolates contribute to resistance gene circulation and spillover.

### 3.6 Correlation analysis of ESBL genes and antibiotic resistance profiles

**Figure 6:**
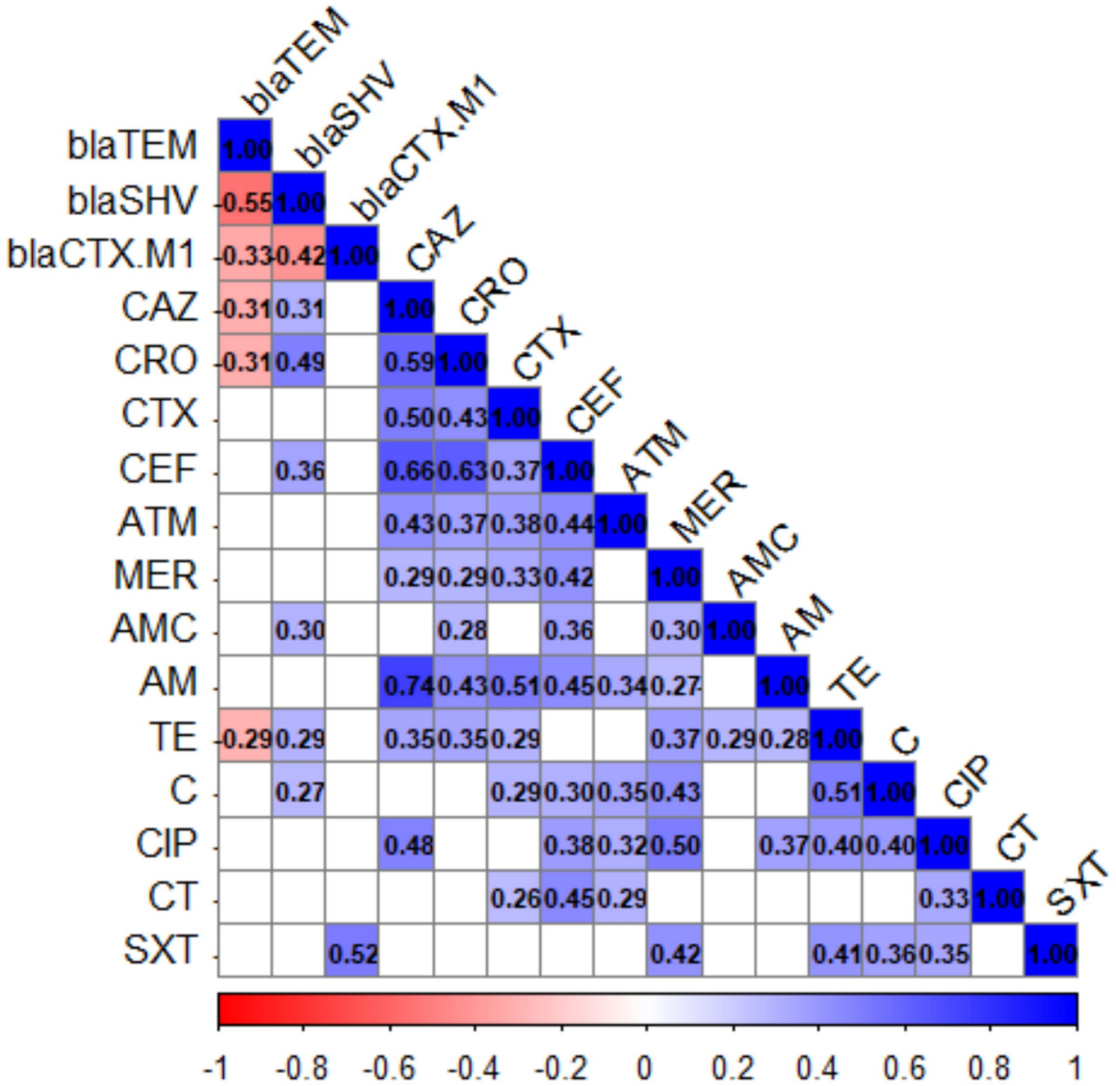
Correlation analysis of ESBL genes and antibiotic resistance profiles among 56 ESBL-producing *Escherichia coli* and *Klebsiella pneumoniae* isolates. Positive and negative correlations are denoted by the red and blue color boxes, respectively. The correlation coefficient’s (r) numerical value is equivalent to the color’s intensity. Boxes with non-significant correlations were left empty, and correlations were calculated at *p* < 0.05.

Correlation analysis between resistance genes (*blaTEM*, *blaSHV*, *blaCTX-M1*) and antibiotic susceptibility profiles revealed several statistically significant associations (Figure 6 and Supplementary Table S8).

A strong negative correlation was observed between *blaTEM* and *blaSHV* (*r* = −0.546, *p* = 1.34 × 10^−5^), indicating that the presence of *blaTEM* is inversely associated with *blaSHV* carriage. Similarly, *blaTEM* showed moderate negative correlations with *blaCTX-M1* (*r* = −0.331, *p* = 0.013), CAZ (*r* = −0.310, *p* = 0.020), and CRO (*r* = −0.313, *p* = 0.019). In contrast, *blaSHV* demonstrated positive correlations with several β-lactams, including CRO (*r* = 0.490, *p* = 1.26 × 10^−4^), CAZ (*r* = 0.305, *p* = 0.022), and CEF (*r* = 0.359, *p* = 0.0066). Notably, *blaCTX-M1* was positively correlated with SXT (*r* = 0.516, *p* = 4.7 × 10^−5^), suggesting a potential co-selection mechanism.

Among antibiotics, the strongest associations were observed between cephalosporins themselves. For example, CAZ was strongly correlated with CRO (*r* = 0.593, *p* = 1.45 × 10^−6^), CTX (*r* = 0.504, *p* = 7.36 × 10^−5^), and CEF (*r* = 0.658, *p* = 3.58 × 10^−8^). Likewise, AM resistance was very strongly associated with CAZ (*r* = 0.740, *p* = 7.11 × 10^−11^). Interestingly, resistance of quinolones (CIP) also showed significant positive correlations with multiple β-lactams (e.g., CAZ: *r* = 0.480, *p* = 1.80 × 10^−4^; CEF: *r* = 0.385, *p* = 0.0034), highlighting possible cross-resistance or co-resistance phenomena.

## 4 Discussion

The dissemination of *E. coli* and *K. pneumoniae* across the animal–human–environment interface represents a major One Health challenge, particularly given their ability to acquire and disseminate resistance traits such as ESBLs. Understanding the baseline prevalence of these organisms and the seasonal factors influencing their distribution is essential for assessing the risk of antimicrobial-resistance spread. The present study recovered in total 385 *E. coli* and 68 *K. pneumoniae* isolates from poultry, dairy cows, farm environment, farm workers, and hospitalized patients to assess seasonal impact on prevalence of ESBL-traits.

First, the prevalence analysis of isolates showed clear seasonal differences in dairy cattle with higher rates detected in fall compared to winter. This may be attributed to changes in temperature, humidity, and farm management practices that favor bacterial survival and transmission (Balta et al., 2024). Similarly, seasonal fluctuation was observed in farm environment, with higher recovery rates in summer and winter, reflecting the influence of environmental stressors and water contamination on bacterial persistence. In contrast, farm workers consistently carried a high prevalence of *E. coli* across seasons, indicating persistent occupational exposure and highlighting the role of humans as reservoirs and potential disseminators within the farm setting. This is greatly aligned with a study in Scotland, which reported that *E. coli* shedding in cattle is seasonally associated with warmer months and reflecting the seasonality of human infections (Ogden et al., 2004).

*K. pneumoniae* prevalence followed a distinct pattern, showing generally lower rates across all samples from different host groups but with significant variation in environmental samples and among dairy cows. This highlights the combined influence of both season and source on its occurrence. These findings are consistent with a review study from Germany, which emphasized the environmental health impact of *K. pneumoniae*, noting its significant resistance to disinfectants and frequent detection in farm-related environmental matrices such as water and sewage (Wareth and Neubauer, 2021). It has been also recently demonstrated that food chain-animals serve as a major reservoir for *E. coli*, while *K. pneumoniae* is less prevalent but more commonly found in environmental samples (Tsitsos et al., 2025).

Interestingly, hospitalized patients did not exhibit clear seasonal trends. This is likely reflecting their continuous exposure to hospital-associated risk factors such as close contact with other carriers rather than external environmental conditions. Similarly, poultry isolates remained relatively stable across seasons which may be driven by the hygiene and biosafety measures and antibiotics use.

Our findings in general revealed source-specific and seasonal differences in bacterial distributions, suggesting that both environmental and management factors contribute to shaping the prevalence of *E. coli* and *K. pneumoniae* organisms. This provides a baseline for subsequent ESBL phenotype and genotype characterization.

Phenotypic and genotypic ESBL analysis revealed notable seasonal and host-associated variations in the prevalence of ESBL-producing *E. coli* and *K. pneumoniae*. In poultry, ESBL- *E. coli* remained relatively stable between summer and fall but slightly increased in winter, whereas *K. pneumoniae* showed a marked seasonal fluctuation, being absent in fall but reaching its highest prevalence in winter. In dairy cows, ESBL-producing *E. coli* were observed in summer and fall but disappeared in winter, suggesting a possible link to management practices or antimicrobial use during warmer months.

Environmental isolates showed seasonal fluctuating in ESBL detection with *K. pneumoniae* more prevalence in winter. Contaminated environments are rich with commensals such as *E. coli* and other *Enterobacteriaceae*, which often carry plasmids, integrons, and transposons that facilitate resistance genes mobilization. Seasonal changes in temperature and humidity may act as stressors that support bacterial persistence and promote genetic exchange. Such ecological pressures, combined with excessive input of antibiotic residues from farming practices, likely create hotspots that facilitate the transfer of ESBL genes among bacterial populations (Larsson and Flach, 2022). This was highlighted in number of studies demonstrated that farm animals and their surrounding environments served as reservoirs to ESBL-producers (Kamaruzzaman et al., 2020) and (Badr et al., 2022). Similarly, environmental sources have been identified as significant contributors to the dissemination of ESBLs in sub-Saharan Africa, especially among *E. coli* isolates (Olaitan et al., 2025). Comparable observations have been reported in recent studies from Brazil (Requena-Castro et al., 2024) and New Zealand (Gray et al., 2025), where surface waters were considered major hotspots for ESBL-producing bacteria.

In farm workers, ESBL- producing *E. coli* prevalence peaked in summer, followed by a slight decrease in fall and complete absence in winter. This seasonal pattern along with the observed increase in the farm environment, suggests a potential risk of zoonotic transmission during warmer months through direct contact, contaminated surfaces, airborne routes, vectors such as flies, and environmental sources like slurry and dairy farm effluents (Dahms et al., 2015). Notably, hospitalized patients exhibited the highest prevalence in fall and summer compared to winter, highlighting the potential for seasonal amplification of resistant strains in clinical settings. Comparably, seasonal and high incidence of ESBL- *E. coli* and *Klebsiella species* among hospitalized patients in German was reported in warmer months (Kaier et al., 2010). Another study also highlighted the seasonal variations of ESBL-carriage among the general Dutch population, which were associated with the warmer months (Wielders et al., 2020).

These results corroborate the influence of seasonal and ecological factors on the dissemination of ESBL genes, with *E. coli* predominating in fall across most sources, while *K. pneumoniae* was more frequently associated with winter, particularly in poultry and environmental samples. This is consistent with the observations of Pak and King (2022), who demonstrated that low temperatures and wind contribute to increased antibiotic resistance and the environmental spread of resistant microbes. A German study also identified environmental and anthropogenic factors particularly seasonal variations, extreme weather events, and cattle density as significant determinants influencing the occurrence of ESBL/*AmpC*-producing *E. coli* in wildlife (Günther et al., 2022). The complete absence of ESBL-producing *K. pneumoniae* in fall among the tested hosts may be attributed to seasonal climatic conditions. In particular, local temperature can strongly influence the persistence of ESBL-carrying plasmids in the environment and affect how efficiently these plasmids are transferred between bacteria. Plasmid stability varies not only between bacterial species but also between strains of the same species, and temperature can influence this stability. For example, Yang et al. reported that some plasmids (such as *blaKPC-IncF* and *blaNDM-IncX3*) were most stable at 25–30 °C and became less stable at 37 °C (Yang et al., 2024). Other experimental work in *E. coli* has shown that plasmid loss is lower at 20 °C than at 37 °C (Wein et al., 2019). In addition, some conditions may limit the spread of ESBL plasmids. Haweky et al. surveyed ESBL strain and plasmid transmission over one year in a hospital setting and found that ESBL plasmid transmission events were generally rare (Hawkey et al., 2022).

The distribution of ESBL genes among *E. coli* and *K. pneumoniae* isolates demonstrated clear seasonal variation by host and environments in current one health setting, although some resistance traits remained stable. In summer, *E. coli* isolates from poultry, dairy, and the environment were dominated by *blaTEM* (100, 75, and 50%, respectively), a pattern that persisted into the fall where poultry and dairy isolates remained universally positive (100%). Contrarily, human *E. coli* showed no *blaTEM* in summer but displayed substantial carriage in fall (42.85%), together with *blaSHV* as the most frequent gene (57.1%). Environmental *E. coli* also shifted between seasons, harboring *blaCTX-M*1 predominantly in fall after being associated with both *blaTEM* (50%) and *blaCTX-M* group 1 (50%) in summer. Winter presented a distinct profile, with *blaTEM* disappearing from *E. coli* across all sources but emerging in *K. pneumoniae* from poultry (50%) and environment (100%). At the same time, *E. coli* in winter became enriched by *blaSHV* (poultry 100%, humans 100%, environment 66.6%) while *blaCTX-M* group 1 was confined to environmental *E. coli* (33.3%) and poultry *K. pneumoniae* (50%). Comparably, a study conducted in El-Sharkia Governorate between December 2019 and April 2021 demonstrated that *blaTEM* and *blaSHV* were the most frequently detected ESBL genes in poultry (Salem et al., 2023). The seasonal occurrence of *blaSHV*, particularly its predominance in humans and its fluctuating detection in poultry and environmental isolates during winter and fall, mirrors its previously reported dominance across multiple reservoirs including humans, livestock, and poultry in northern Egypt (Nossair et al., 2022). In the contrary, *blaCTX-M* group 9 was consistently absent across all three seasons, suggesting limited introduction or persistence of this lineage in the studied ecosystem.

These patterns indicate that *blaTEM* is a stable trait in poultry *E. coli*, while both *blaSHV* and *blaCTX-M* group 1 fluctuate seasonally across hosts and niches, possibly reflecting differences in antimicrobial usage, environmental pressures, and bacterial adaptability. Likewise, a study on Dutch poultry farm environments showed that the vast majority of *E. coli* isolates harbored *blaCTX-M-1*, *blaSHV*, and *blaTEM* (Blaak et al., 2015) Additionally, a meta-analysis from sub-Saharan Africa, which included subgroup analysis by sample source, identified animals as the predominant reservoir of ESBL-producing *E. coli*, with *blaCTX-M* reported as the most prevalent ESBL gene (Olaitan et al., 2025).

Hierarchical analysis of ESBL-gene distribution and antibiotic-resistance profiles showed clustering of isolates belonging to different hosts. The results showed that *blaTEM* tends to be inversely associated with other ESBL genes (*blaSHV*, *blaCTX-M1*) and certain cephalosporins, whereas *blaSHV* and *blaCTX-M1* are significantly linked with cephalosporins and other non-β-lactam antibiotics. The clustering of correlations among cephalosporins indicates that resistance to these agents may be strongly co-selected, possibly reflecting the underlying genetic determinants of ESBL production.

The association of ESBL genes with resistance to antibiotic classes not inhibited by ESBL genes has previously been demonstrated in several studies (Badr et al., 2022; Nossair et al., 2022; Yousfi et al., 2016). Our data reported a strong correlation between *blaCTX-M1and* SXT resistance which suggests cross resistance and co-location of multiple resistance traits on mobile genetic elements. The sul1 gene, which confers resistance to sulfonamides, is commonly carried within class 1 integrons, genetic elements known for capturing and expressing multiple resistance gene cassettes (Jiang et al., 2019). Detection of mobile genetic elements such as integrons in ESBL- isolates from feces of farm and domestic animals has been linked to phenomenon of extensive resistance to non- cephalosporin antibiotics (Ejaz et al., 2021). Several studies have shown that class 1 integrons containing sul1 can occur on the same mobile genetic elements such as ISCR1-associated transposons that also harbor *blaCTX-M* genes (Mendonça et al., 2007; Shahid et al., 2012). This close physical linkage means that the resistance determinants can move together on a single plasmid or integron. As a result, the observed co-resistance pattern likely reflects a shared genetic background that enables both traits to be maintained and transmitted simultaneously.

This finding and previous studies identified ESBL-producers as a One Health threat both as pathogen and disseminators to multi-resistance traits (Tello et al., 2022). ESBL-producing isolates showed multidrug-resistant patterns, including resistance to colistin, which is a last-resort antibiotic. Several studies have also reported a link between ESBL production and colistin resistance in *E. coli* and *K. pneumoniae*(Lay et al., 2021; Hide et al., 2024). For example, Trongjit et al. (2022) found that ESBL-producing bacteria from pigs could transfer both *bla* genes and the colistin-resistance gene *mcr-3* to *Salmonella.* This suggests that some strains may carry both resistance traits together (Trongjit et al., 2022).

A key limitation of this study is the lack of seasonal antimicrobial use data, which may have influenced the observed variations in ESBL prevalence. Additionally, not all known ESBL genes were tested, so the presence of other resistance determinants cannot be excluded. In a future study, whole genome sequencing of the isolates will be conducted to provide deeper molecular insights and clarify the roles of gene mobilization, plasmids, and integrons.

## 5 Conclusion

This study investigated seasonal influence on the prevalence and dissemination of ESBL-producing *E. coli* and *K. pneumoniae* within a One Health context. The results revealed a notable seasonal pattern associated with both animal and human hosts, as well as with specific bacterial species, while also highlighting the farm environment as an important reservoir for ESBL producers. Furthermore, a correlation was observed between harboring ESBL genes and resistance to non-β-lactam antibiotics, rather than to β-lactams themselves. These findings highlight the significance of ESBL-producing bacteria as both pathogens and carriers, driving bacterial evolution while promoting cross-resistance and gene mobilization within shared ecological niches. This underscores the need for seasonally tailored antimicrobial interventions and integrated One Health surveillance, including enhanced hygiene measures during high-risk seasons and closer monitoring of plasmid-carrying strains to limit the spread of ESBL genes.

## Data Availability

The original contributions presented in the study are included in the article/Supplementary material, further inquiries can be directed to the corresponding author.
