# Supplementary material for: Seasonal prevalence of extended-spectrum β-lactamase–producing bacteria in food-chain animals, humans, and the surrounding environment in Fayoum governorate: a one health approach
Source: Front Microbiol. 2026 Feb 4;17:1726798. doi: 10.3389/fmicb.2026.1726798 (PMC12913390; doi:10.3389/fmicb.2026.1726798)
Supplement: Supplementary file 1 [file Table_1.docx]

| **Seasons** | **Summer** | | | **Fall** | | | **Winter** | | | **P.value**  **different seasons and same species** | **P.value**  **different seasons and same species** |
| --- | --- | --- | --- | --- | --- | --- | --- | --- | --- | --- | --- |
| **Analysis** | **VIETK** | | | **VIETK** | | | **VIETK** | | |  |  |
|  | **Samples**  **(n)** | *E. coli*  n (%) | *K. pneumoniae*  n (%) | **Samples**  **(n)** | *E. coli*  n (%) | *K. pneumoniae*  n (%) | **Samples**  **(n)** | *E. coli*  n (%) | *K. pneumoniae*  n (%) | *E. coli* | *K. pneumoniae* |
| **Poultry** | 1130: Pooled into113 | 25(22.1) | 8(7.07) | 860 pooled into86 | 17(19.76) | 3(3.48) | 900 pooled into90 | 10(11.11) | 4(4.44) | 0.112 | 0.490 |
| **Dairy cows** | 320 | 47(14.68) | 10(3.1) | 366 | 61(16.66) | 3(0.81) | 340 | 15(4.41) | 4(1.17) | 0.000* | 0.043* |
| **Environments** | 195 | 38(19.48) | 16(8.20) | 192 | 18(9.37) | 0 | 196 | 39(19.89) | 9(4.59) | 0.007* | 0.000* |
| **Farm workers** | 58 | 26(44.82) | 7(12.06) | 35 | 19(54.28) | 0 | 25 | 13(52) | 0 | 0.643 | 0.021* |
| **Hospitalized patients (fecal samples)** | 50 | 7(14) | 1(2) | 40 | 5(12.5) | 1(2.5) | 55 | 16(29.09) | 0 | 0.065 | 0.527 |
| **Total** | 736 | 143(19.42) | 42(5.70) | 719 | 120(16.68) | 7(0.97) | 705 |  |  |  |  |
| **P.value**  **For each M.O in same season and different sources** |  | 0.000* | 0.016* |  | 0.000* | 0.063 |  | 0.000* | 0.045* |  |  |

**Table S1. Prevalence rate of *E. coli* and *K. pneumoniae* among the different hosts across three seasons based**

**on VITEK.**

* Statistically significant at P.value <0.05
