# Supplementary material for: Seasonal prevalence of extended-spectrum β-lactamase–producing bacteria in food-chain animals, humans, and the surrounding environment in Fayoum governorate: a one health approach
Source: Front Microbiol. 2026 Feb 4;17:1726798. doi: 10.3389/fmicb.2026.1726798 (PMC12913390; doi:10.3389/fmicb.2026.1726798)
Supplement: Supplementary file 2 [file Table_2.docx]

| **Analysis** | **Phenotypic and Genotypic ESBL- producers %** | | | | | | **P.value**  **different seasons and same species** |
| --- | --- | --- | --- | --- | --- | --- | --- |
| **Seasons** | **Summer** | | **Fall** | | **Winter** | |  |
|  | **Isolates**  **(N)** | **ESBL**  **Isolates**  **N** (%) | **Isolates**  **(N)** | **ESBL**  **Isolates**  **N** (%) | **Isolates**  **(N)** | **ESBL**  **Isolates**  **N** (%) |  |
| **Poultry** | ***E. coli***  **(25)** | ESBL *E. coli*  4(16) | ***E. coli* (17)** | ESBL *E. coli*  2 (11.76) | ***E. coli* (10)** | ESBL *E. coli*  2(20) | .843 |
|  | ***K. pneumoniae***  **(8)** | ESBL *K. pneumoniae*  0 | ***K. pneumoniae* (3)** | ESBL *K. pneumoniae*  0 | ***K. pneumoniae* (4)** | ESBL *K. pneumoniae*  2(50) | .042* |
| **Dairy cows** | ***E. coli* (47)** | ESBL *E. coli*  4(8.5) | ***E. coli* (61)** | ESBL *E. coli*  8(13.11) | ***E. coli* (15)** | ESBL *E. coli*  0 | .289 |
|  | ***K. pneumoniae* (10)** | ESBL *K. pneumoniae*  1(10) | ***K. pneumoniae* (3)** | ESBL *K. pneumoniae*  0 | ***K. pneumoniae***  **(4)** | ESBL *K. pneumoniae*  0 | .689 |
| **Environments** | ***E. coli* (38)** | ESBL *E. coli*  4(10.5) | ***E. coli* (18)** | ESBL *E. coli*  1(5.5) | ***E. coli***  **(39)** | ESBL *E. coli*  3(7.7) | .804 |
|  | ***K. pneumoniae* (16)** | ESBL *K. pneumoniae*  1(6.25) | ***K. pneumoniae* (0)** | ESBL *K. pneumoniae*  0 | ***K. pneumoniae***  **(9)** | ESBL *K. pneumoniae*  3(33.3) | .116 |
| **Farm workers** | ***E. coli* (26)** | ESBL *E. coli*  6(23.08) | ***E. coli* (19)** | ESBL *E. coli*  4(21.05) | ***E. coli***  **(13)** | ESBL *E. coli*  0 | .172 |
|  | ***K. pneumoniae* (7)** | ESBL *K. pneumoniae*  3(42.8) | ***K. pneumoniae* (0)** | ESBL *K. pneumoniae*  0 | ***K. pneumoniae***  **0** | ESBL *K. pneumoniae*  0 | .^a^ |
| **Hospitalized patients (fecal samples)** | ***E. coli* (7)** | ESBL *E. coli*  3(42.8) | ***E. coli* (5)** | ESBL *E. coli*  3(60) | ***E. coli***  **(16)** | ESBL *E. coli*  2(12.5) | .076 |
|  | ***K. pneumoniae* (1)** | ESBL *K. pneumoniae*  0 | ***K. pneumoniae***  **(1)** | ESBL *K. pneumoniae*  0 | ***K. pneumoniae***  **(0)** | ESBL *K. pneumoniae*  0 | .^a^ |
| **Human (Farm workers and Hospitalized patients** | ***E. coli* (33)** | ESBL *E. coli*  9(27.3) | ***E. coli* (24)** | ESBL *E. coli*  7(29.2) | ***E. coli***  **(29)** | ESBL *E. coli*  2(6.9) |  |
|  | ***K. pneumoniae* (8)** | ESBL *K. pneumoniae*  3(37.5) | ***K. pneumoniae***  **(1)** | ESBL *K. pneumoniae*  0 | ***K. pneumoniae***  **(0)** | ESBL *K. pneumoniae*  0 |  |
| **Total** | ***E. coli* (143)** | ESBL *E. coli* 21(14.68) | ***E. coli* (120)** | ESBL *E. coli* 18(15) | ***E. coli***  **(93)** | ESBL *E. coli*  7(7.5) |  |
|  | ***K. pneumoniae* (42)** | ESBL *K. pneumoniae* 5(11.9) | ***K. pneumoniae* (7)** | ESBL *K. pneumoniae* **(0)** | ***K. pneumoniae***  **(17)** | ESBL *K. pneumoniae*  5(29.4) |  |
| **P. value**  **For E.coli in same season and different sources** | .096 |  | .039* |  | .279 |  |  |
| **P. value**  **For *K. pneumoniae*** **in same season and different sources** |  | .087 |  | .^a^ |  | .279 |  |
