# Supplementary material for: Seasonal prevalence of extended-spectrum β-lactamase–producing bacteria in food-chain animals, humans, and the surrounding environment in Fayoum governorate: a one health approach
Source: Front Microbiol. 2026 Feb 4;17:1726798. doi: 10.3389/fmicb.2026.1726798 (PMC12913390; doi:10.3389/fmicb.2026.1726798)
Supplement: Supplementary file 3 [file Table_3.docx]

| **ESBL-phenotypes detection using disk diffusion method** | | | | | | | | | | | | | | | |
| --- | --- | --- | --- | --- | --- | --- | --- | --- | --- | --- | --- | --- | --- | --- | --- |
| **ESBL- Screening antibiotics (Resistance %)** | | | | | | | | | | | | | | | |
| **Antibiotics** | **Dairy (No. =4)** | | | **Poultry (No.=4)** | | | **Environment (No. =4)** | | | **Human (No. =9)** | | | **Total (No. =21)** | | |
|  | **R** | **I** | **S** | **R** | **I** | **S** | **R** | **I** | **S** | **R** | **I** | **S** | **R** | **I** | **S** |
|  | NO. (%) | NO. (%) | NO.  (%) | NO. (%) | NO. (%) | NO  (%) | NO. (%) | NO. (%). | NO. (%) | NO. (%). | NO. (%). | NO. (%) | NO. (%). | NO. (%). | NO. (%) |
| **MEM (10 µg)** | 0 | 3(75) | 1(25) | 1(25) | 2(50) | 1(25) | 0 | 3(75) | 1(25) | 2(22.2) | 4(44.4) | 3(33.3) | 3(14.2) | 12(57.1) | 6(28.5) |
| **AMC (20 µg /10 µg)** | 0 | 0 | 4(100) | 3(75) | 0 | 1(25) | 2(50) | 0 | 2(50) | 4(44.4) | 1(11.1) | 4(44.4) | 9(42.8) | 1(4.7) | 11(52.3) |
| **AM (10 µg)** | 4(100) | 0 | 0 | 4(100) | 0 | 0 | 3(75) | 0 | 1(25) | 9(100) | 0 | 0 | 20(95.2) | 0 | 1(4.7) |
| **TE (30 µg)** | 2(50) | 0 | 2(50) | 4(100) | 0 | 0 | 3(75) | 0 | 1(25) | 8(88.9) | 1(11.1) | 0 | 17(80.9) | 0 | 4(19) |
| **C (30 µg)** | 2(50) | 0 | 2(50) | 3(75) | 0 | 1(25) | 3(75) | 0 | 1(25) | 6(66.6) | 1(11.1) | 2(22.2) | 14(66.6) | 1(4.7) | 6(28.5) |
| **CIP (5 µg)** | 4(100) | 0 | 0 | 3(75) | 1(25) | 0 | 3(75) | 1(25) | 0 | 6(66.6) | 3(33.3) | 0 | 16(76) | 5(23) | 0 |
| **CT (10 µg)** | 3(75) | 0 | 1(25) | 4(100) | 0 | 0 | 4(100) | 0 | 0 | 9(100) | 0 | 0 | 20(95.2) | 0 | 1(4.7) |
| **SXT (1.25 µg /23.75 µg)** | 2(50) | 0 | 2(50) | 4(100) | 0 | 0 | 2(50) | 0 | 2(50) | 6(66.6) | 0 | 3(33.3) | 14(66.6) | 0 | 7(33.3) |

**Table S3. Antibiotic resistance-pattern of ESBL-producing *E. coli* during summer season**

R: resistant; I: intermediate; S: sensitive
