# Supplementary material for: Seasonal prevalence of extended-spectrum β-lactamase–producing bacteria in food-chain animals, humans, and the surrounding environment in Fayoum governorate: a one health approach
Source: Front Microbiol. 2026 Feb 4;17:1726798. doi: 10.3389/fmicb.2026.1726798 (PMC12913390; doi:10.3389/fmicb.2026.1726798)
Supplement: Supplementary file 4 [file Table_4.docx]

| **ESBL-phenotypes detection using disk diffusion method** | | | | | | | | | | | | | | | |
| --- | --- | --- | --- | --- | --- | --- | --- | --- | --- | --- | --- | --- | --- | --- | --- |
| **ESBL- Screening antibiotics (Resistance %)** | | | | | | | | | | | | | | | |
| **Antibiotics** | **Dairy (No. =8)** | | | **Poultry (No .=2)** | | | **Environment (No. =1)** | | | **Human (No. =7)** | | | **Total (No. =18)** | | |
|  | **R** | **I** | **S** | **R** | **I** | **S** | **R** | **I** | **S** | **R** | **I** | **S** | **R** | **I** | **S** |
|  | NO. (%) | NO. (%) | NO  (%) | NO. (%) | NO. (%) | NO. (%) | NO. (%) | NO. (%). | NO. (%) | NO. (%). | NO. (%). | NO. (%) | NO. (%). | NO. (%). | NO. (%) |
| **MEM (10 µg)** | 0 | 2(25) | 6(75) | 2(100) | 0 | 0 | 0 | 0 | 1(100) | 4(57.1) | 3(42.8) | 0 | 4(22) | 5(27) | 9(50) |
| **AMC (20 µg /10 µg)** | 3(37.5) | 0 | 5(62.5) | 1(50) | 0 | 1(50) | 1(100) | 0 | 0 | 3(42.8) | 0 | 4(57.1) | 8(44.4) | 0 | 10 (55.5) |
| **AM (10 µg)** | 6(75) | 1(12.5) | 1(12.5) | 2(100) | 0 | 0 | 1(100) | 0 | 0 | 7(100) | 0 | 0 | 16(88) | 1(5.5) | 1(5.5) |
| **TE (30 µg)** | 2(25) | 0 | 6(75) | 2(100) | 0 | 0 | 0 | 0 | 1(100) | 6(85.7) | 0 | 1(14.3) | 10(55.5) | 0 | 8(44.4) |
| **C (30 µg)** | 3 (37.5) | 0 | 5(62.5) | 2(100) | 0 | 0 | 0 | 0 | 1(100) | 4(57.1) | 0 | 3(42.8) | 9(50) | 0 | 9(50) |
| **CIP (5 µg)** | 2(25) | 3(37.5) | 3(37.5) | 2(100) | 0 | 0 | 1(100) | 0 | 0 | 7(100) | 0 | 0 | 12(66.6) | 3(16.6) | 3(16.6) |
| **CT (10 µg)** | 5(62.5) | 0 | 3(37.5) | 1(50) | 0 | 1(50) | 1(100) | 0 | 0 | 7(100) | 0 | 0 | 14(77.7) | 0 | 4(22) |
| **SXT (1.25 µg /23.75 µg)** | 1(12.5) | 0 | 7(87.5) | 2(100) | 0 | 0 | 1(100) | 0 | 0 | 4(57.1) | 0 | 3(42.8) | 8(44.4) | 0 | 10(55.5) |

**Table S4. Antibiotic resistance-pattern of ESBL-producing *E. coli* during fall season**

R: resistant; I: intermediate; S: sensitive
