# Supplementary material for: Seasonal prevalence of extended-spectrum β-lactamase–producing bacteria in food-chain animals, humans, and the surrounding environment in Fayoum governorate: a one health approach
Source: Front Microbiol. 2026 Feb 4;17:1726798. doi: 10.3389/fmicb.2026.1726798 (PMC12913390; doi:10.3389/fmicb.2026.1726798)
Supplement: Supplementary file 7 [file Table_7.docx]

| **ESBL-phenotypes detection using disk diffusion method** | | | | | | | | | | | | | | | | |
| --- | --- | --- | --- | --- | --- | --- | --- | --- | --- | --- | --- | --- | --- | --- | --- | --- |
| **ESBL- Screening antibiotics (Resistance %)** | | | | | | | | | | | | | | | | |
| **Antibiotics** | **Dairy (No. =0)** | | | **Poultry (No.=2)** | | | **Environment (No. =3)** | | | | **Human Isolates (No. =0)** | | | **Total (No. =5)** | | |
|  | **R** | **I** | **S** | **R** | **I** | **S** |  | **R** | **I** | **S** | **R** | **I** | **S** | **R** | **I** | **S** |
|  | NO. (%) | NO. (%) | NO  (%) | NO. (%) | NO. (%) | NO(%) |  | NO. (%) | NO. (%). | NO. (%) | NO. (%). | NO. (%). | NO. (%) | NO. (%). | NO. (%). | NO. (%) |
| **MEM (10 µg)** | 0 | 0 | 0 | 1(50) | 0 | 1(50) |  | 0 | 0 | 3(100) | 0 | 0 | 0 | 1(20) | 0 | 4(80) |
| **AMC (20 µg /10 µg)** | 0 | 0 | 0 | 1(50) | 0 | 1(50) |  | 0 | 0 | 3(100) | 0 | 0 | 0 | 1(20) | 0 | 4(80) |
| **AM (10 µg)** | 0 | 0 | 0 | 2(100) | 0 | 0 |  | 2(66) | 1(33) | 0 | 0 | 0 | 0 | 4(80) | 1(20) | 0 |
| **TE (30 µg)** | 0 | 0 | 0 | 1(50) | 0 | 1(50) |  | 1(33) | 0 | 2(66) | 0 | 0 | 0 | 2(40) | 0 | 3(60) |
| **C (30 µg)** | 0 | 0 | 0 | 1(50) | 0 | 1(50) |  | 1(33) | 0 | 2(66) | 0 | 0 | 0 | 2(40) | 0 | 3(60) |
| **CIP (5 µg)** | 0 | 0 | 0 | 1(50) | 0 | 1(50) |  | 0 | 0 | 3(100) | 0 | 0 | 0 | 1(20) | 0 | 4(80) |
| **CT (10 µg)** | 0 | 0 | 0 | 2(100) | 0 | 0 |  | 1(33) | 0 | 2(66) | 0 | 0 | 0 | 3(60) | 0 | 2(40) |
| **SXT (1.25 µg /23.75 µg)** | 0 | 0 | 0 | 1(50) | 0 | 1(50) |  | 0 | 1(33) | 2(66) | 0 | 0 | 0 | 1(20) | 1(20) | 3(60) |

**Table S7. Antibiotic resistance-pattern of ESBL-producing *K. pneumoniae*** **during winter season**
