# Supplementary material for: Seasonal prevalence of extended-spectrum β-lactamase–producing bacteria in food-chain animals, humans, and the surrounding environment in Fayoum governorate: a one health approach
Source: Front Microbiol. 2026 Feb 4;17:1726798. doi: 10.3389/fmicb.2026.1726798 (PMC12913390; doi:10.3389/fmicb.2026.1726798)
Supplement: Supplementary file 8 [file Table_8.docx]

**Table. S8. Correlation analysis between resistance genes and antibiotic resistance profiles**

| **Var1** | **Var2** | **r** | **p_value** | **Direction** | **Strength** | **Significance** |
| --- | --- | --- | --- | --- | --- | --- |
| blaSHV | blaTEM | -0.54616 | 1.34E-05 | Negative | Strong | *** |
| blaCTX.M1 | blaTEM | -0.33068 | 0.012802 | Negative | Moderate | * |
| blaCTX.M1 | blaSHV | -0.41629 | 0.001417 | Negative | Moderate | ** |
| AMC | blaSHV | 0.297796 | 0.025809 | Positive | Weak | * |
| blaTEM | CAZ | -0.31027 | 0.019951 | Negative | Moderate | * |
| blaSHV | CAZ | 0.305499 | 0.022044 | Positive | Moderate | * |
| ATM | CAZ | 0.431933 | 0.000887 | Positive | Moderate | *** |
| AM | CAZ | 0.740137 | 7.11E-11 | Positive | Very strong | *** |
| blaTEM | CRO | -0.31278 | 0.018922 | Negative | Moderate | * |
| blaSHV | CRO | 0.49002 | 0.000126 | Positive | Moderate | *** |
| CAZ | CRO | 0.593251 | 1.45E-06 | Positive | Strong | *** |
| CEF | CRO | 0.62675 | 2.37E-07 | Positive | Strong | *** |
| ATM | CRO | 0.368734 | 0.005168 | Positive | Moderate | ** |
| AMC | CRO | 0.282748 | 0.034732 | Positive | Weak | * |
| AM | CRO | 0.43313 | 0.000855 | Positive | Moderate | *** |
| CAZ | CTX | 0.504455 | 7.36E-05 | Positive | Strong | *** |
| CRO | CTX | 0.432367 | 0.000875 | Positive | Moderate | *** |
| CEF | CTX | 0.368485 | 0.0052 | Positive | Moderate | ** |
| ATM | CTX | 0.379061 | 0.003964 | Positive | Moderate | ** |
| AM | CTX | 0.50763 | 6.52E-05 | Positive | Strong | *** |
| C | CTX | 0.293087 | 0.028367 | Positive | Weak | * |
| CT | CTX | 0.26469 | 0.048678 | Positive | Weak | * |
| blaSHV | CEF | 0.358771 | 0.006622 | Positive | Moderate | ** |
| CAZ | CEF | 0.657972 | 3.58E-08 | Positive | Strong | *** |
| ATM | CEF | 0.44066 | 0.000676 | Positive | Moderate | *** |
| AMC | CEF | 0.358695 | 0.006634 | Positive | Moderate | ** |
| AM | CEF | 0.445268 | 0.000584 | Positive | Moderate | *** |
| C | CEF | 0.300623 | 0.024368 | Positive | Moderate | * |
| AM | ATM | 0.336924 | 0.011112 | Positive | Moderate | * |
| CAZ | MER | 0.288139 | 0.031279 | Positive | Weak | * |
| CRO | MER | 0.285197 | 0.033127 | Positive | Weak | * |
| CTX | MER | 0.334252 | 0.01181 | Positive | Moderate | * |
| CEF | MER | 0.420071 | 0.001268 | Positive | Moderate | ** |
| AMC | MER | 0.297259 | 0.02609 | Positive | Weak | * |
| AM | MER | 0.267432 | 0.046306 | Positive | Weak | * |
| C | MER | 0.433441 | 0.000847 | Positive | Moderate | *** |
| CIP | MER | 0.500404 | 8.59E-05 | Positive | Strong | *** |
| blaTEM | TE | -0.29383 | 0.027951 | Negative | Weak | * |
| blaSHV | TE | 0.293285 | 0.028255 | Positive | Weak | * |
| CAZ | TE | 0.349749 | 0.008235 | Positive | Moderate | ** |
| CRO | TE | 0.348688 | 0.008446 | Positive | Moderate | ** |
| CTX | TE | 0.292119 | 0.028918 | Positive | Weak | * |
| MER | TE | 0.372935 | 0.004644 | Positive | Moderate | ** |
| AMC | TE | 0.2868 | 0.032109 | Positive | Weak | * |
| AM | TE | 0.27804 | 0.038001 | Positive | Weak | * |
| C | TE | 0.508153 | 6.38E-05 | Positive | Strong | *** |
| CIP | TE | 0.39871 | 0.002337 | Positive | Moderate | ** |
| SXT | TE | 0.410206 | 0.00169 | Positive | Moderate | ** |
| blaSHV | C | 0.271707 | 0.042796 | Positive | Weak | * |
| ATM | C | 0.35264 | 0.007684 | Positive | Moderate | ** |
| CAZ | CIP | 0.480176 | 0.00018 | Positive | Moderate | *** |
| CEF | CIP | 0.384606 | 0.003426 | Positive | Moderate | ** |
| ATM | CIP | 0.316149 | 0.01761 | Positive | Moderate | * |
| AM | CIP | 0.369525 | 0.005065 | Positive | Moderate | ** |
| C | CIP | 0.396611 | 0.002477 | Positive | Moderate | ** |
| CEF | CT | 0.454994 | 0.000426 | Positive | Moderate | *** |
| ATM | CT | 0.294081 | 0.02781 | Positive | Weak | * |
| CIP | CT | 0.330398 | 0.012883 | Positive | Moderate | * |
| blaCTX.M1 | SXT | 0.515831 | 4.73E-05 | Positive | Strong | *** |
| MER | SXT | 0.422782 | 0.00117 | Positive | Moderate | ** |
| C | SXT | 0.360046 | 0.006418 | Positive | Moderate | ** |
| CIP | SXT | 0.345623 | 0.00908 | Positive | Moderate | ** |
